# Detection of allele-specific expression in spatial transcriptomics with spASE

**DOI:** 10.1186/s13059-024-03317-4

**Published:** 2024-07-08

**Authors:** Luli S. Zou, Dylan M. Cable, Irving A. Barrera-Lopez, Tongtong Zhao, Evan Murray, Martin J. Aryee, Fei Chen, Rafael A. Irizarry

**Affiliations:** 1 Department of Biostatistics, Harvard T.H. Chan School of Public Health, Boston, MA 02115 USA; 2 Department of Data Sciences, Dana-Farber Cancer Institute, Boston, MA 02215 USA; 3 Broad Institute of Harvard and MIT, Cambridge, MA 02142 USA; 4 Department of Electrical Engineering and Computer Science, MIT, Cambridge, MA 02139 USA

**Keywords:** Spatial transcriptomics, Allele-specific expression

## Abstract

Spatial transcriptomics technologies permit the study of the spatial distribution of RNA at near-single-cell resolution genome-wide. However, the feasibility of studying spatial allele-specific expression (ASE) from these data remains uncharacterized. Here, we introduce spASE, a computational framework for detecting and estimating spatial ASE. To tackle the challenges presented by cell type mixtures and a low signal to noise ratio, we implement a hierarchical model involving additive mixtures of spatial smoothing splines. We apply our method to allele-resolved Visium and Slide-seq from the mouse cerebellum and hippocampus and report new insight into the landscape of spatial and cell type-specific ASE therein.

**Supplementary Information:**

The online version contains supplementary material available at 10.1186/s13059-024-03317-4.

## Background

In diploid organisms, allele-specific expression (ASE) refers to the imbalanced expression of the two parental alleles for a given gene. ASE has been well-studied in the context of epigenetic phenomena such as genomic imprinting and X-chromosome inactivation (XCI) [1–3], where expression from one allele is silenced. Spatial patterns of ASE have long been observed as a consequence of XCI in female organisms, where the random silencing of either the maternal or paternal X-chromosome in early development is passed to daughter cells, resulting in visible clusters of ASE [4–6]. By contrast, although studies in bulk and single-cell RNA-sequencing data have revealed widespread variability in ASE throughout the autosome across tissues and cell types [7–20], relatively little is known about the prevalence of spatial ASE therein.

Spatial transcriptomics technologies now provide the opportunity to study spatial ASE patterns genome-wide. For example, Slide-seq [21, 22] has high resolution which enables near-single-cell quantification of ASE with 2D spatial information. However, for most technologies, including both Slide-seq and Visium, measurement locations can potentially source transcripts from multiple cells and cell types. Furthermore, these data are limited by highly sparse read counts in comparison to bulk or single-cell sequencing technologies, which is exacerbated by the requirement that reads align uniquely to one allele. In addition, cell type, which drives the majority of variability observed in single-cell data, is highly correlated with spatial location, especially in solid tissue [23]. Therefore, it is important to distinguish between spatial and cell type-specific ASE, which could arise from and contribute to distinct underlying biological mechanisms.

Several statistical and computational methods have been developed for studying ASE in bulk and single-cell RNA-seq data [24–32]. Some focus on estimating allele-specific transcriptional bursting kinetics for individual genes in homogeneous populations of cells [15, 30, 31]. Here, we instead focus on the problem of estimation and inference for the maternal allele probability *p* for a given gene across 2D space, and we consider how *p* may vary with cell type. To model *p* in bulk and single-cell RNA-seq, multiple methods have used a beta-binomial framework, which can flexibly account for overdispersion from unknown technical and biological variability [26–28, 32]. An additional advantage of this model is that it can be parameterized as a generalized linear model (GLM) [33, 34], allowing for maximum likelihood estimation of *p* while incorporating covariates of interest such as cell type. One statistical challenge that has not yet been addressed is how to account for cell type mixtures, particularly when cell types can have varying rates of gene expression as well as distinct epigenomic profiles which can lead to preferential expression of one allele.

The issue of estimating smooth functions from sparsely sampled data has been well-studied [35–40], and multiple solutions have been developed and implemented as computational methods [41, 42]. In the case of allele-specific spatial transcriptomics data, although the read count measured at individual spatial coordinates may be low, smoothing spline methods can increase power by leveraging information from local neighborhoods of spots. Generalized additive models are GLMs that incorporate smoothing splines into a regression framework, enabling estimation of the smooth spatial function as well as hypothesis testing for spatial functions deviating from a constant [39, 42].

Here, we present spASE, a statistical and computational framework for detecting ASE in spatial transcriptomics while accounting for cell type mixtures. Building on the beta-binomial strategy used in previous ASE models, we introduce a mixture framework that estimates the contribution from each cell type to maternal and paternal allele counts at each spot, calculated based on cell type proportions and differential expression. Our method enables modeling of the maternal allele probability spatial function both across and within cell types as well as the ability to distinguish between cell type-specific and within cell type spatial ASE. We generated Slide-seq and Visium data from an F1 hybrid mouse model, and we use spASE to estimate the prevalence of different types of ASE, including overall (bulk) ASE, cell type-specific ASE, and spatial ASE. Furthermore, we use spASE to generate high resolution spatial maps of X-chromosome ASE and identify a set of genes escaping XCI. We further demonstrate the utility of spASE in generating high resolution spatial maps of X-chromosome ASE across all samples, and for visualization of estimated spatial patterns.

## Results

### A beta-binomial framework for modeling allele-specific expression in spatial transcriptomics

We generated Visium and Slide-seq data from four genetically identical female F1 hybrid (CAST x 129) mice (Additional file 1: Figs. S1, S2, Table S1, the “Methods” section). This hybrid strain is ideal for assessing allele-specific expression (ASE) as it contains a high rate of genetic differences within transcripts, including polymorphisms and indels, that can be uniquely assigned to one of the two parental inbred strains. In total, we sequenced two mouse cerebellums, one with Visium and one with Slide-seq, and three mouse hippocampuses with Slide-seq. We called cell types on each sample using RCTD [23] in doublet mode for the Slide-seq samples and full mode for Visium (Additional file 1: Figs. S3-S6).

These data revealed two primary statistical challenges associated with analyzing ASE from spatial transcriptomics. The first challenge is that spatial and cell type effects can be confounded, particularly in solid tissues such as the mouse cerebellum (Additional file 1: Fig. S7a,b). Measurement locations, here termed *spots*, can potentially source transcripts from multiple cell types, making it difficult to detect and control for cell type effects on allelic expression. A second challenge is that for technologies that have high resolution, such as Slide-seq (Additional file 1: Fig. S7b), we often observe spots with low counts and high overdispersion. This overdispersion is sometimes referred to in single-cell literature as “transcriptional bursting,” as it leads to “bursts” of monoallelic expression, even for autosomal genes that have no prior knowledge of exhibiting allele-specific expression, such as *Aldoc* in the mouse cerebellum (Additional file 1: Fig. S7c,d). This extra variability is particularly present in spatial transcriptomics when the resolution of measurements approaches single cells, e.g., more so in Slide-seq as compared to Visium (Additional file 1: Figs. S7e,f, S8).

**Fig. 1:**
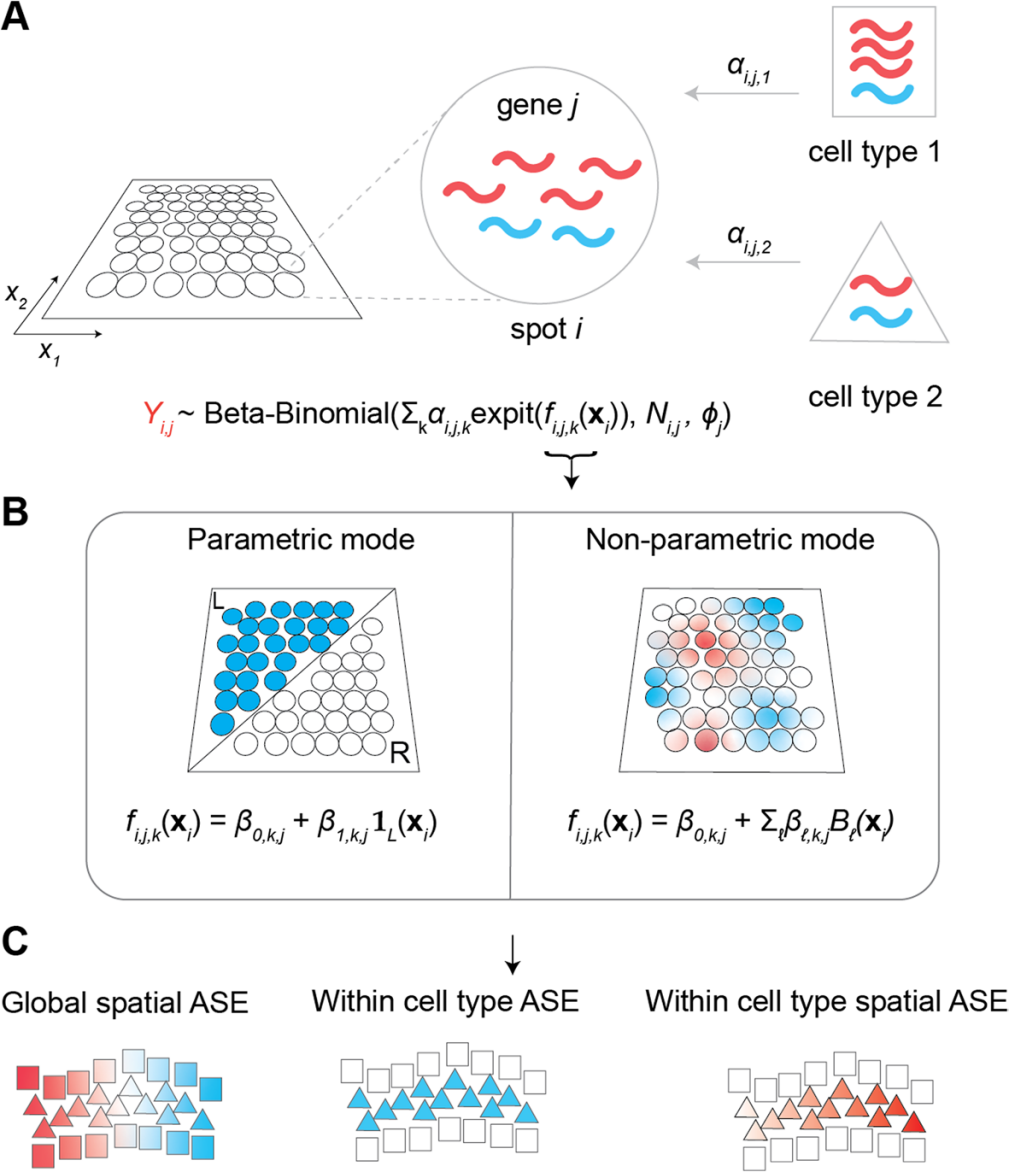
Schematic of the spASE method for detecting allele-specific expression (ASE) in spatial transcriptomics while accounting for mixtures. **a** Given a 2D spatial transcriptomics data set, we assume that for each gene *j*, each spot *i* can potentially source transcripts from multiple cell types. Each cell type can potentially have a different rate of expression for gene *j* and could also have a different cell type-specific maternal (red) or paternal (blue) bias for gene *j*. In this example, cell type 1 has a higher level of expression as well as a maternal bias, whereas cell type 2 has lower expression and no bias. The weights $\alpha$ determine the contribution of each cell type to the transcripts on spot *i*. **b** spASE can be run in two modes: parametric and non-parametric. Parametric mode is designed for hypothesis testing, e.g., testing whether or not there is a difference in ASE between two regions. Non-parametric mode is for when no prior hypothesis exists about ASE, and the goal is to flexibly estimate a smooth function across space. **c** spASE can determine whether or not a gene exhibits significant ASE, either spatially, within cell type, or both. Notation: $Y_{i,j}$ represents the maternal allele counts at spot *i* for gene *j*. $\textbf{x}_i=(x_{1,i}, x_{2,i})$ are the 2D spatial coordinates of spot *i*. $\alpha _{i,j,k}$ are the cell type-specific weights. $\textbf{1}_L(\cdot )$ is the indicator function for membership in set *L*. $B_{\ell }$ denotes the $\ell ^{\text {th}}$ spline basis function

We therefore developed a statistical framework to model and account for both these sources of variability. Specifically, we developed a beta-binomial model for mixtures of cell types and provide a flexible approach to estimation, inference, and visualization of ASE in spatial transcriptomics (Fig. 1). We denote the counts from the maternal allele for spot *i* and gene *j* with $Y_{i,j}$ and assume it follows the distribution:

1 $$\begin{aligned} Y_{i,j}{} & {} \sim \text {Beta-Binomial}(p_{i,j}, N_{i,j}, \phi _j) \nonumber \\ p_{i,j}{} & {} = \sum \limits _{k=1}^K \alpha _{i,j,k} \, \text {expit} \left( \beta _{0,k,j} + \sum \limits _{\ell =1}^L \beta _{\ell ,k,j}\gamma _{i,\ell }\right) , \end{aligned}$$

where we parameterize the beta-binomial distribution in terms of mean probability, total read count, and a gene-specific overdispersion (Additional file 1: Supplemental Notes). Specifically, $p_{i,j}$ is the mean probability that a transcript from gene *j* is from the maternal allele; $N_{i,j}$ is the total read count, summing both alleles; $\phi _j$ is a gene-specific overdispersion term ranging from 0 to 1 that accounts for biological and technical variability not explained by binomial sampling; $\alpha _{i,j,k}$ are cell type-specific weights; and the $\gamma _{i,\ell }$ represent any potential user-defined covariates of interest, such as categorical regions or a smoothing spline basis function evaluated at continuous spatial coordinates (see below).

Our model for the expected maternal allele probability, $p_{i,j}$, is a weighted sum over the contributions at each spot for each gene from all cell types $k \in \{1,\ldots ,K\}$ present at that spot. To properly formulate the weights, $\alpha _{i,j,k}$, we considered the possibility that there could be cell type-specific differences in both the rate of gene expression as well as the allelic bias. Thus, we designed the weights to adjust the expected maternal probability contribution at spot *i* from cell type *k* by (1) the rate of expression for gene *j* in cell type *k* and spot *i* and (2) the proportion of transcripts at spot *i* that are expected to be attributable to cell type *k* (Fig. 1a, see the “Methods” section). We pre-compute these weights from the cell type weights at each spot obtained through a cell type decomposition algorithm (e.g., RCTD) and the cell type-specific gene expression rates estimated using a differential expression algorithm (e.g., C-SIDE [43], the “Methods” section). This allows us estimate and perform inference on the cell type-specific covariates, $\beta$.

In practice, the expression within the expit function in the model for the mean can take a variety of forms, depending on the biological hypothesis of interest (Fig. 1b). For example, we may be interested in testing for allelic differences between two pre-defined regions; in this case, we test for significance of the coefficients $\beta$. Alternatively, if we have no prior hypothesis about allelic variation, we can choose to flexibly model a 2D function across space using a non-parametric smoothing spline approach. The latter option is particularly useful as it allows for estimation of the maximum likelihood smooth maternal allele probability function, either across or within cell type (Fig. 1c, the “Methods” section).

### spASE detects ASE in Visium and Slide-seq while accounting for cell type mixtures

**Fig. 2:**
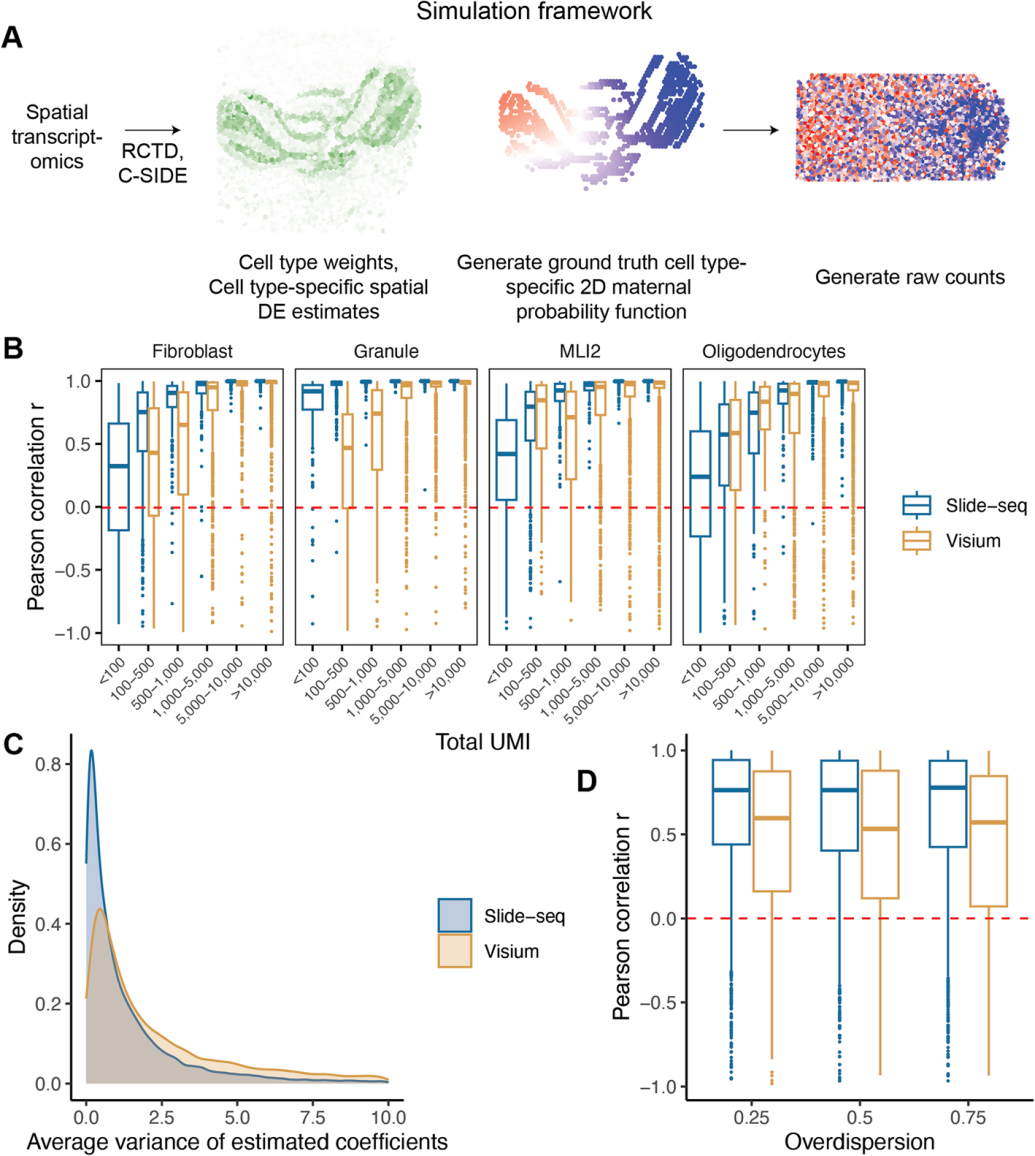
Simulation framework and results comparing estimated 2D maternal probability functions to ground truth. **a** Simulation framework schematic. We used the Visium and Slide-seq data generated in this study and ran RCTD to get cell type weights at each spot as well as C-SIDE to get cell type-specific gene expression rates at each spot. **b** Pearson correlation *r* between the estimated 2D maternal allele probability function and the ground truth maternal allele probability function, binned by the total UMI for that simulation condition ($N_{i,j}$ in the model). Panel title indicates the cell type that had the true cell type-specific ASE pattern. **c** Density plots of the variance of the estimated coefficients (logit scale) across all simulations. **d** Same *r* as (**b**), except only for total UMI 100-500, including all cell types, and binned by ground truth overdispersion values

We first conducted simulations to test the ability of spASE to accurately estimate smooth, non-parametric 2D spatial functions in the presence of cell type mixtures (Fig. 2). Using the real Visium and Slide-seq data, we estimated gene- and cell type-specific proportions and differential expression (DE) at each spot. We generated random linear combinations of spline basis functions as ground truth 2D spatial patterns and sampled raw counts from the ground truth distribution at each spot (Fig. 2a). We then fit the spASE model and computed the RMSE of the coefficient estimation (Additional file 1: Fig. S9) as well as the correlation between the estimated 2D maternal probability function values and the ground truth (Fig. 2b).

Overall, we found that the higher resolution from Slide-seq resulted in both lower RMSE of coefficient estimation and higher $R^2$ of the estimated maternal probability functions (Fig. 2b), particularly at lower total UMI counts per gene. We attributed this difference to a reduction in variance of the estimated coefficients given the higher density of points sampled in the function (Fig. 2c) as well as lower variance in the cell type-specific DE estimates in Slide-seq as compared to Visium (Additional file 1: Fig. S10). Even at 100–500 counts, correlation was still weakly positive, with the inner quartile range of the boxplots generally lying above $r=0.5$. This motivated our selection of the threshold of $2^7$ (128 total counts) in analyses using real data as well as using confidence intervals over the 2D surface to assess the variance in the estimated 2D spatial function and account for cases where the density of counts for a particular gene was not high enough to make an accurate estimate. There was also a noticeable cell type effect; in particular, cell types with a broader spatial distribution across the slice, such as granule cells in the cerebellum, were easier to estimate at lower UMI values than cells with more limited spatial distributions. We also varied the ground truth level of overdispersion in the data and found that higher overdispersion resulted in higher variability, especially for the Visium samples (Fig. 2d), which was also likely due to the lower sample size in Visium.

Next, we fit the beta-binomial model on the real Slide-seq and Visium data. We tested for significant (1) overall maternal or paternal bias, (2) within cell type bias, (3) overall spatial pattern, and (4) within cell type spatial pattern, using a false discovery rate threshold of $q<0.01$, and report the prevalence of significant ASE in each of these categories (Tables 1 and 2).

**Table 1:** Number of genes detected as significant ($q < 0.01$) in the F1 hybrid (CAST x 129) mouse cerebellum in Visium and Slide-seq from two female mouse brains

Category	Slide-seq (mouse 3)	Visium (mouse 4)	Overlap
No significant ASE	6235 (53)	8971 (162)	5425 (30)
Overall maternal bias	720 (157)	502 (112)	196 (81)
Overall paternal bias	947 (7)	672 (12)	300 (5)
Within cell type maternal bias	306 (62)	9 (2)	5 (2)
Within cell type paternal bias	416 (4)	14 (0)	7 (0)
Overall spatial pattern	8 (19)	2 (0)	1 (0)
Within cell type spatial pattern	0 (6)	0 (0)	0 (0)
Total *n* genes	8304 (225)	10,147 (286)	7599 (204)

“Overlap” column indicates number of genes that were detected as significant in both mice (i.e., intersection of the numbers for mouse 3 and mouse 4 for each row). Note that genes can belong to more than one category, with the exception of the “no significant ASE” category, which is exclusive. Numbers are formatted as # autosomal genes (# X-chromosome genes). The total number of genes for each sample is based on using a filtering threshold of at least 128 spots with non-zero counts per gene for “overall” or at least 128 spots per gene per cell type for “within cell type”

**Table 2:** Number of genes detected as significant ($q < 0.01$) in the F1 hybrid (CAST x 129) mouse hippocampus in Slide-seq from three female mouse brains

Category	Mouse 1	Mouse 2	Mouse 3	Ovl. (1-2)	Ovl. (1-3)
No significant ASE	4961 (104)	2242 (5)	6530 (16)	2008 (3)	4249 (4)
Overall maternal bias	349 (30)	176 (55)	623 (208)	93 (11)	179 (29)
Overall paternal bias	456 (9)	180 (1)	834 (1)	109 (1)	254 (1)
Within cell type maternal bias	104 (3)	24 (15)	250 (61)	18 (1)	64 (1)
Within cell type paternal bias	151 (9)	22 (0)	307 (0)	19 (0)	101 (0)
Overall spatial pattern	18 (17)	1 (0)	67 (0)	1 (0)	9 (0)
Within cell type spatial pattern	0 (0)	0 (0)	0 (0)	0 (0)	0 (0)
Total *n* genes	5866 (159)	2609 (61)	8309 (225)	2560 (59)	5698 (150)

Overlap (Ovl.) is calculated between mice 1 and 2 and mice 1 and 3. Genes can belong to more than one category, with the exception of the “no significant ASE” category, which is exclusive. Numbers are formatted as # autosomal genes (# X-chromosome genes). The total number of genes for each sample is based on using a filtering threshold of at least 128 spots with non-zero counts per gene for “overall” or at least 128 spots per gene per cell type for “within cell type”

We first compared the overall estimates of maternal and paternal bias for each gene across all samples (Additional file 1: Fig. S11). Consistent with prior knowledge, we identified a small set of genes exhibiting strong maternal and paternal bias across all hippocampus and cerebellum samples, including *Meg3*, *Impact*, and *Peg3*, for which our estimated direction of ASE bias was consistent with previous reports as maternal (*Meg3*) and paternal (*Impact*, *Peg3*) imprinted genes [44]. We compared these estimates to a previously published list of 383 imprinted genes in mice (not specific to the brain) [45] and found that 91 of them were also detected as imprinted in our data (Additional file 2: Table S2, Additional file 3: Table S3). We also used a resource which contains previous knowledge on the direction of ASE effect for 138 genes (not specific to the brain) [44] and found that, of the 35 genes estimated to have ASE in our data set, 26 of them (18 paternal and 8 maternal) agreed with the previous direction of effect (Additional file 2: Table S2, Additional file 3: Table S3). A large number of genes were also consistently subtly, but not significantly, biased either maternally or paternally across samples. The vast majority of genes had an estimated maternal probability close to 0.5. Interestingly, the agreement between the hippocampus and cerebellum in mouse 3 was much higher than the agreement among the hippocampus samples or cerebellum samples ($R^2=0.91$ compared to $R^2 \in [0.32,0.69]$), suggesting higher ASE variability across mice than between two brain regions of the same mouse (Additional file 1: Fig. S11b).

We next compared results across significant spatial and non-spatial ASE gene categories between the Visium and Slide-seq cerebellum samples (Table 1). Of the 7599 autosomal genes detected in both samples, the majority (5425 or 71%) had no significant ASE detected in either sample, for a concordance of roughly $87\%$ between the two mice. 496 autosomal genes genes, or 6.5%, had a significant maternal or paternal bias in both samples. For cell type-specific ASE, we noticed that the Slide-seq sample had a much higher number of genes detected with a bias than the Visium sample (722 compared to 23). More genes were also detected as having an overall spatial pattern in Slide-seq compared to Visium (8 vs. 2). Finally, six X-chromosome genes were detected as having a spatial ASE pattern within cell type in the Slide-seq sample, while none were detected in the Visium sample. Thus, despite having a lower average number of allele-resolved counts per spot than Visium (Additional file 1: Table S1), the higher resolution and density of measurement spots in Slide-seq affords higher power to detect both cell type-specific and spatial ASE.

Similarly, we compared the prevalence of ASE in the different categories across three Slide-seq mouse hippocampus samples (Table 2). Samples with higher sequencing depth and longer read lengths generally had more total genes detected in each category (Additional file 1: Table S1). Notably, in both the cerebellum and hippocampus, agreement across samples was modest, hovering around 50–70% overlap in gene sets for each category.

### spASE separates cell type-driven spatial ASE from within cell type spatial ASE in the mouse hippocampus and cerebellum

**Fig. 3:**
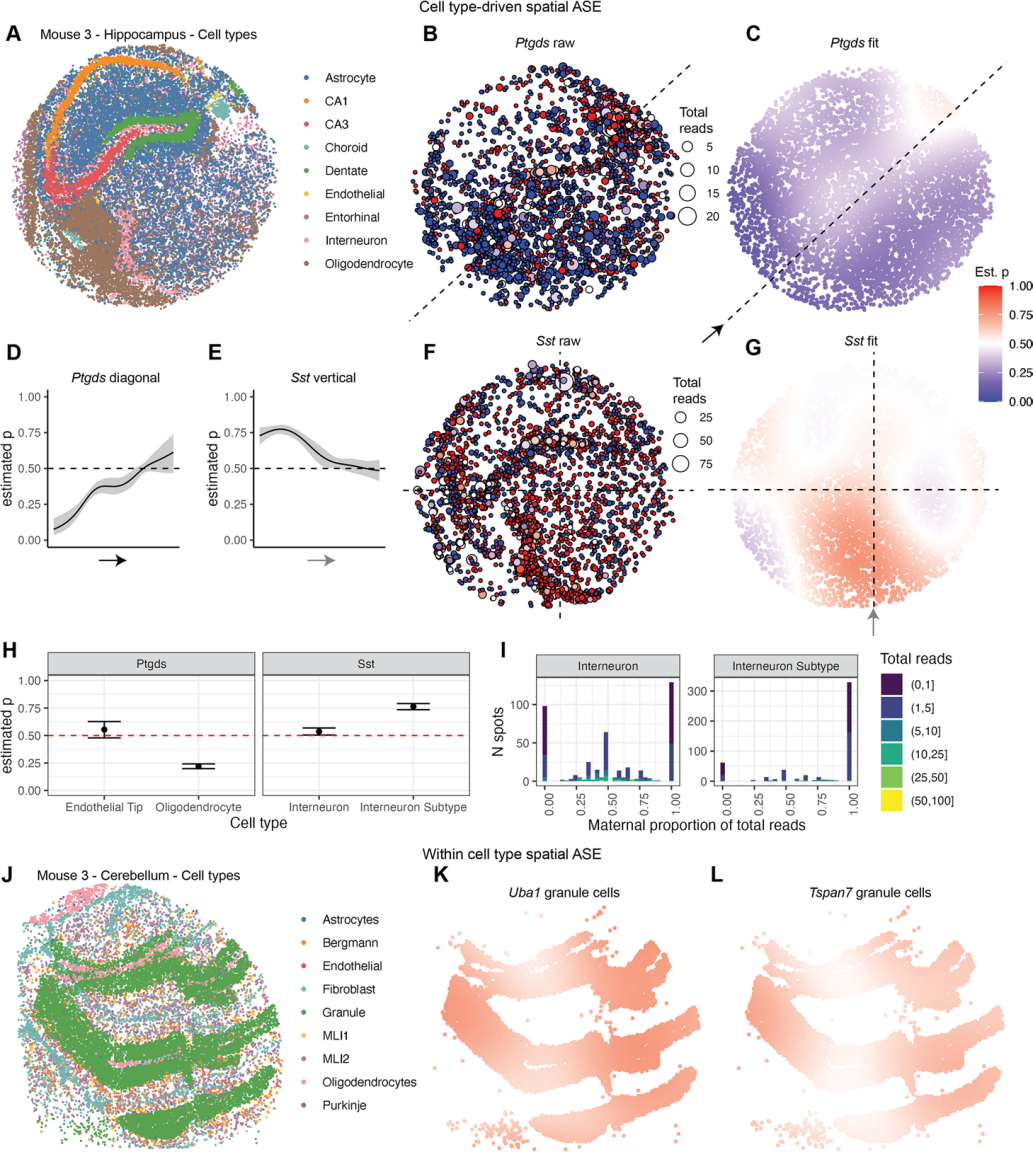
spASE distinguishes between cell type driven and within cell type spatial ASE in the mouse hippocampus and cerebellum. **a** Cell type map of the hippocampus from mouse 3. **b** Raw maternal allele fractions for each spot with non-zero expression of *Ptgds* in the hippocampus from mouse 3. Spot size corresponds to the total number of allele-resolved reads (total reads) at that location. Color corresponds to the fraction of reads at that spot that were maternal; red being maternal, blue being paternal, and white being 50/50. **c** Estimated maternal allele probability function computed by spASE across cell types. **d** Estimate and confidence interval cross-section along the diagonal of **c**. **e** Estimate and confidence interval cross-section along the vertical dashed line in **g**. **f**, **g** Same as **b**–**c**, but for *Sst*. **h** Estimate and confidence interval from the intercept-only (within cell type) spASE model for *Ptgds* and *Sst* by cell type. Only cell types with sufficient reads measured at spots containing that cell type are included. **i** Distributions of the maternal proportion of total reads per spot for *Sst* by cell type, colored by the total number of reads per spot. **j** Cell type map for the cerebellum from mouse 3. **k** Same as **c** but for *Uba1* and only plotted at the location of granule cell singlets. **l** Same as **k** but for *Tspan7*

We next assessed whether our method could accurately differentiate between cell type-driven and within cell type spatial ASE in real data. Cell type is often confounded with space, particularly in the hippocampus, in which cell types are concentrated in discrete spatial areas within the 2D slice (Fig. 3). Without controlling for cell type, we observed a considerable number of autosomal genes with a significant overall spatial ASE pattern: 18 genes in mouse 1 and 67 in mouse 3, which had higher coverage, with 9 genes in common (Table 2, Additional file 4: Table S4). Here, we further make a distinction in terminology for genes exhibiting cell type-specific ASE, cell type-driven spatial ASE, or within cell type spatial ASE. *Cell type-specific ASE* refers to ASE that is only present in one particular cell type. *Cell type-driven spatial* ASE refers to a spatial bias in ASE that could be due to cell type-specific ASE, or it could also be driven by a higher rate of expression for a particular gene relative to other cell types, even if the ASE is not cell type-specific. *Within cell type spatial ASE* refers to spatial ASE detectable within a cell type which is not necessarily cell type-specific.

For example, consider the gene *Ptgds*, which exhibited significant overall spatial ASE pattern of a general paternal bias with a small region of biallelic expression in the mouse hippocampus (Fig. 3a–c). These regions corresponded to the localization of two major cell types which highly expressed *Ptgds*: oligodendrocytes and endothelial cells. When using spASE to estimate the effect size of ASE within cell types, we found that oligodendrocytes had a strong paternal bias in expression, whereas endothelial cells had strong biallelic expression (Fig. 3h). When using the full spASE model controlling for cell type, *Ptgds* was not significant for having a within cell type spatial ASE effect. Thus, we conclude that *Ptgds* exhibits a cell type-driven spatial ASE effect due to the contrast between the biallelic expression in endothelial cells, which are localized to a small portion of the region, and high paternal expression in oligodendrocytes, which encompass a broader area of the sample. We wondered what potential mechanism could be driving the paternal bias in oligodendrocyte expression (Fig. 3h). Integrating single-cell ATAC-seq data and single-cell RNA-seq atlas data, we found a likely mechanism by which an oligodendrocyte-specific enhancer for *Ptgds* may have a strain-specific SNP which alters an oligodendrocyte-specific TF binding site (Additional file 1: Fig. S12).

We also discovered a cell type-driven spatial effect in *Sst* in the hippocampus. In particular, we noticed a strong maternal bias in the bottom-middle region of the puck, corresponding to a cluster of interneurons outside of the main hippocampal formation in the thalamus (Fig. 3e–g). Indeed, when we fit the intercept-only spASE model and examined the estimated maternal probabilities within cell type, we found that this interneuron subtype had a higher maternal estimated maternal probability that was further from 0.5 than other interneurons (Fig. 3h). This bias was also visible in the distributions pf raw maternal proportion of total reads per cell type (Fig. 3i). Thus, the localization of this interneuron subtype to a small region of the puck, combined with its high, strongly maternal expression of *Sst*, was the main driver of the spatial pattern in *Sst*.

Cell types within the cerebellum also aggregate in distinct spatial regions; however, depending on the slice, the main granule layer comprises a wide spatial range of densely packed cells, spanning a larger area and density than most other cell types (Fig. 3h). We found that the only genes detected as having a significant spatial ASE pattern within cell type were six highly expressed X-chromosome genes in granule cells (Additional file 5: Table S5). In particular, we saw that the wide span of granule cells was large enough to capture the subtle spatial X-chromosome ASE pattern present in the cerebellum of Mouse 3 (Fig. 3i,j).

### spASE generates high resolution spatial maps of the allele-specific X-chromosome landscape in the mouse hippocampus and cerebellum

Finally, we used non-parametric spASE to estimate smooth 2D maternal allele probability functions for X-chromosome genes in all our samples. In addition to testing for significance, the non-parametric mode allows for varying the degrees of freedom of the smoothing spline basis functions to create higher resolution maps of the ASE patterns depending on gene coverage.

We first examined the fitted 2D maternal allele functions for all X-chromosome genes individually for each of the five mice (Additional file 1: Figs. S13-S17). For all samples, the majority of X-chromosome genes were estimated to have a similar smooth pattern within a sample. We observed some slight differences which could be attributed to lack of UMI sampled in some genes, leading to higher variance in the estimates.

We then looked for any genes potentially escaping X-chromosome inactivation (XCI). We compared the estimated maternal probability functions for a previously published list of 14 XCI escape genes in the mouse brain [46] and found that 11 (including *Xist*) were highly expressed enough in our data to enable estimation of spatial ASE. Of those 11, *Gpm6b* and *Syp* showed evidence of XCI escape in the cerebellum, but not the hippocampus (Additional file 1: Fig. S18). In particular, *Gpm6b* had near-biallelic expression across the slice, and it had a spatial function that was similar, although more attenuated in magnitude, to other X-chromosome genes that did not escape XCI. *Syp* also exhibited near-biallelic expression and exhibited a similar spatial pattern to *Xist* (Additional file 1: Fig. S18). The other genes either had too few counts to confidently estimate a spatial pattern with a small confidence interval, or they appeared to not escape XCI. We also found evidence of XCI escape genes not previously reported; for example, *Morf4l2* showed a strong paternal bias across all five mice and both brain regions (Additional file 1: Figs. S13-S17). In mice 1 and 4, *Tceal3* appeared to have a strong paternal bias, and similarly, *Tceal6* had a pattern that trended closer to biallelic expression than other X-chromosome genes, potentially indicating escape from XCI (Additional file 1: Figs. S13-S17).

**Fig. 4:**
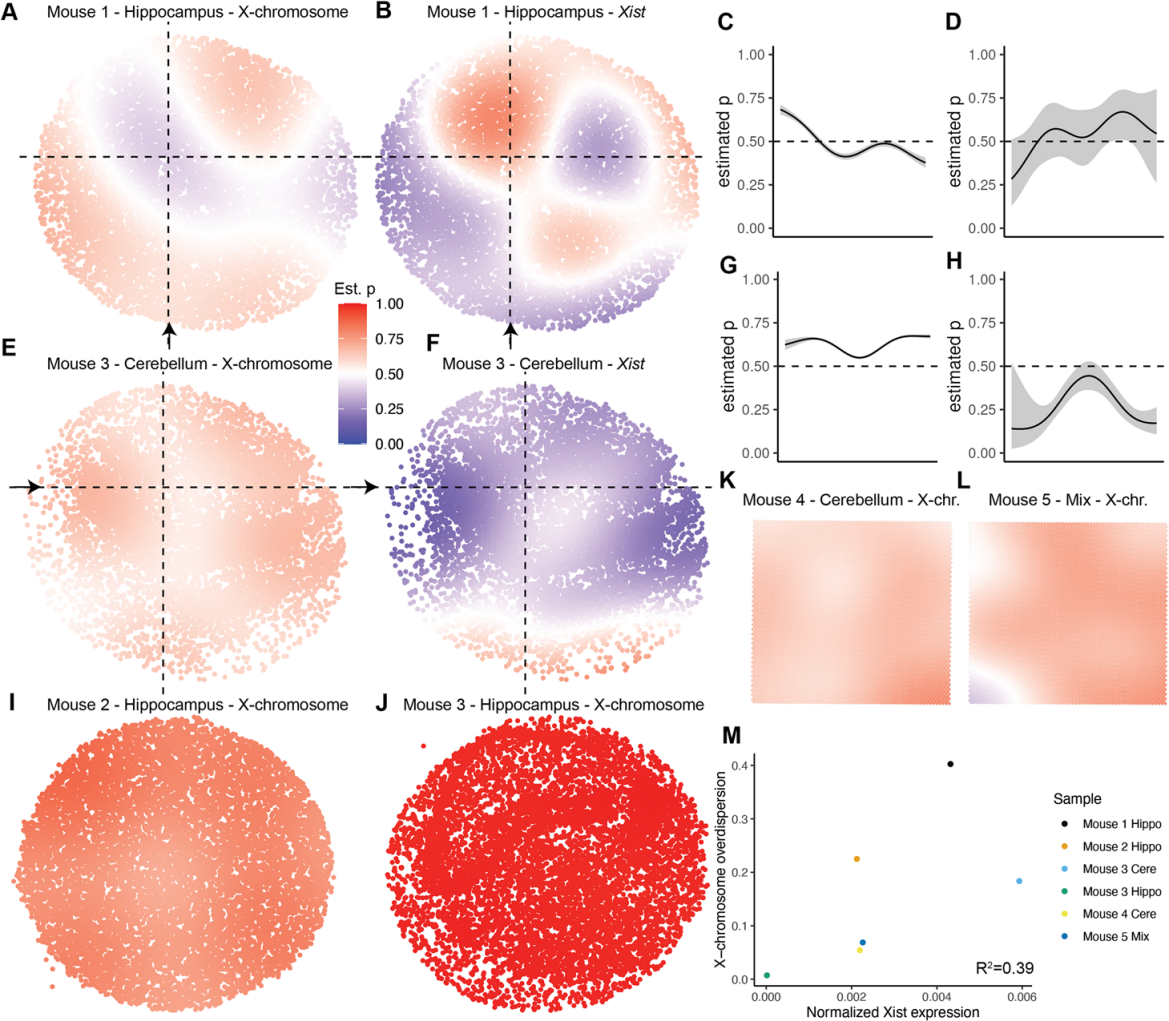
spASE generates high resolution maps of the X-chromosome ASE landscape in the mouse hippocampus and cerebellum from five mice. **a** Estimated maternal allele probability function for the merged X-chromosome profile from the hippocampus of mouse 1. **b** Same as **a**, but for the gene *Xist*. **c** Estimate and confidence interval for the maternal allele probability for all merged X-chromosome genes along the vertical dashed line in a, starting at the arrow in **a** and moving up. **d** Same as **c**, but for *Xist* in **b**. **e**–**h** Same as **a**–**d**, but for the cerebellum of mouse 3. **i**–**l** Same as **a** but for the denoted mice and tissues. **m** Scatterplot showing the merged X-chromosome gene overdispersion plotted against the normalized *Xist* expression for each sample

We then merged all X-chromosome genes (excluding *Xist*) to provide maximum power for estimating the landscape of the X-chromosome in all samples (Fig. 4, Additional file 1: Fig. S19). Overall, we detected considerable heterogeneity in the fitted X-chromosome functions across mice. In the hippocampus of mouse 1, for example, we found distinct regions of clearly maternal and clearly paternal bias, which we confirmed were anti-correlated with regions of maternal and paternal bias in *Xist* (Fig. 4a, b). High coverage from the merged X-chromosome gene profiles allowed for very narrow confidence intervals in the estimate as compared to those for *Xist*, which had larger variance in the estimated maternal probability due to having significantly lower counts (Fig. 4c, d).

Similarly, in the cerebellum of mouse 3, we observed an overall spatial pattern (driven mostly by granule cell expression, as seen in Fig. 3i, j) which was maternally biased towards the left and right edges, but trended towards biallelic near the center (Fig. 4e–h). This pattern was also anti-correlated with *Xist* expression, which again had much lower total coverage resulting in wider confidence intervals. However, we saw that the rest of the samples had a more consistent maternal bias (Fig. 4i–l). In fact, although the cerebellum from mouse 3 overall only exhibited a slight maternal bias, the hippocampus from mouse 3 exhibited a near total silencing of the paternal allele.

We wondered whether this heterogeneity in X-chromosome spatial ASE could be explained by any other genes or factors present in our data set. Interestingly, we found that total *Xist* expression was weakly correlated with the variance in X-chromosome allele probability for each sample, quantified as the estimated overdispersion in the beta-binomial model ($R^2=0.39$, Fig. 4m). In particular, the hippocampus of mouse 3 had nearly 0 *Xist* expression, whereas the cerebellum of mouse 3 and the hippocampus of mouse 1 had considerably higher expression, perhaps indicating dynamic X-inactivation activity.

## Discussion

In summary, we developed spASE, a statistical and computational framework for detecting ASE in spatial transcriptomics while accounting for cell type mixtures. Building on the beta-binomial framework used in previous ASE models [27, 28, 32], we introduce a mixture framework that estimates the contribution from each cell type to maternal and paternal allele counts at each spot, which we calculate based on estimates of cell type identity as well as cell type-specific differential expression. Our method enables modeling of the maternal allele probability spatial function both across and within cell types as well as the ability to distinguish between cell type-driven and within cell type spatial ASE. We used spASE to estimate the prevalence of different types of ASE (overall, within cell type, and spatial) in multiple hippocampus and cerebellum samples from an F1 hybrid mouse model. We further demonstrated the utility of non-parametric spASE in generating high resolution spatial maps of X-chromosome ASE across all samples and for visualizing estimated spatial patterns and their variance at any given location in the spatial 2D function.

Overall, we found modest agreement between previously published imprinted genes and the genes we report here, but many of the genes we found were not in any previously published list of imprinted genes [44, 45]. In particular, only 91 of 383 previously published imprinted genes were detected in our study. However, of the genes that had previously published direction of ASE and that were detected as significant in our analysis, a higher fraction (26 of 35) agreed in the direction of ASE. There are many possible explanations for these differences, including (1) our study was focused on the mouse cerebellum and hippocampus, whereas many studies have not focused on these tissues, and many genes have previously been shown to have imprinting dependent on the tissue of interest [44]; and (2) the genetic background of our mouse model could potentially alter some imprinted directions, particularly with the abundance of SNPs that could affect gene regulatory regions.

Indeed, a limitation of the biological findings presented here is that our experiments only included one mouse strain, CASTx129. Thus, some results, such as the cell type-specific bias for *Ptgds* or the strong maternal bias in the interneuron subtype for *Sst*, may only hold for samples where similar genetic variation exists at the cis-regulating loci. In general, ASE analysis is subject to the available genetic variation that allows for detection of different alleles. We used an F1 hybrid mouse strain which has substantial genetic variation and therefore relatively higher power to detect ASE effects, but such analysis is likely also feasible in other data sets for a smaller set of genes. For example, in a study looking at 10x Chromium, it was shown that estimating ASE was feasible from human scRNA-seq datasets given a large enough library size (average of 150K sequencing reads per cell) [47].

Recently, it has been shown in single cell data that autosomal genes exhibit transcriptional bursting [15, 31], or random monoallelic expression [14], which we model here as overdispersion in the beta-binomial framework. How this bursting, or overdispersion, influences spatial ASE patterns has not yet been studied. Consistent with previous findings, we observed considerable overdispersion in Slide-seq, which has a resolution that is closest to single cell, whereas the count distribution in Visium was more similar to binomial sampling (Additional file 1: Fig. S8). Importantly, however, we did not detect any autosomal genes to have significant within cell type spatial ASE patterns. Thus, we show here that this transcriptional bursting of autosomal alleles is not significantly correlated across space in our data.

Much of X-chromosome biology is still actively being researched [48–50]. Our knowledge of the regulatory networks involved in directing *Xist* expression and ensuring XCI is still incomplete in both mouse and human [48]. Here, we observed that total *Xist* expression was weakly correlated with the amount of spatial variation in the overall X-chromosome gene pattern (Fig. 4m). This result potentially points to a dynamic process of XCI maintenance in adult mice, i.e., less *Xist* RNA present, or captured in sequencing, when the allele is already almost fully silenced.

In addition, our knowledge of the regulatory factors responsible for XCI escape are still unknown [48]. Previous work has shown that random XCI patterns are often skewed away from equal representation of both alleles [51]. Interestingly, we found that *Gpm6b* and *Syp*, two genes previously published to escape XCI in the brain [46], exhibited XCI escape in the cerebellum but not the hippocampus, and many of the previously reported genes did not escape XCI in our data. This could point to tissue- or sample-specific differences in XCI. One limitation was that many of the previously reported genes were not measured at high enough read depth in our data and thus had very wide confidence intervals across the spatial fit (Additional file 1: Fig. S18). However, we did observe that *Morf4l2* consistently escaped XCI across all samples and tissues, showing a strongly paternally biased spatial pattern. The effect was slightly more subtle in the hippocampus from mouse 3, which had a strong maternal bias for all other X-chromosome genes (Additional File 1: Fig. S15). In the cerebellum from mouse 3, *Morf4l2* had a paternal bias but also a region of biallelic expression towards the middle, similar to *Xist*, indicating that its escape from XCI was not ubiquitous. The same was true for *Tceal3* and *Tceal6* in the hippocampus of mouse 1 and the cerebellum of mouse 4 (Additional file 1: Figs. S13, S17); however, these two genes did not seem to escape XCI in mice 2 and 3 (Additional file 1: Figs. S14, S15). *Tceal3* and *Morf4l2* also lie relatively close together on the X-chromosome within 100kb of each other, and *Tceal6* lies slightly over a megabase downstream from *Tceal3*. Thus, there could be some local epigenetic effect allowing these genes to escape XCI in some, but not all samples. In general, we note that the 2D slice gathered for each sample is only a small cross-section of the hippocampus or cerebellum; thus, many samples could have had more X-chromosome genes detected as spatially significant if the field of view were larger.

The reliance on non-parametric smoothing splines to flexibly estimate the maternal allele probability function when no hypothesis is given has both advantages and disadvantages. Advantages include (1) incorporation of the basis functions as covariates into a linear model and thus (2) control over the bias-variance trade-off when choosing degrees of freedom for the spline fit. Disadvantages include (1) no meaningful interpretation of individual coefficients in non-parametric mode and (2) possibly overly smooth estimated spatial functions. Indeed, smoothing splines do not account for sharper boundaries in ASE; for example, although the thin curve of highly expressing endothelial cells partially contributed to the biallelic expression in the center of the hippocampus sample for *Ptgds*, the estimated maternal allele probability function smoothed the thin curve of biallelic expression into a larger area (Fig. 3). In the future, it will be useful to develop methods which more precisely account for both large smooth regions as well as the sharp, discrete cell type regions as we observed in the mouse hippocampus and cerebellum.

## Conclusions

Overall, this work represents an important methodological first step towards using high throughput technologies to characterize the spatial allelome. In simulated and real Slide-seq data from the healthy adult mouse hippocampus and cerebellum, we show that spASE can recover known and unknown cell type-driven and within-cell-type spatial ASE. We additionally show that higher resolution is necessary to have sufficient power to detect cell type-driven effects. Given the difficulty in generating probes that hybridize specifically to different alleles when they could vary by only a few SNPs, we anticipate that sequencing based approaches will continue to be a primary method of measuring ASE transcriptome-wide. We show here that Slide-seq data, when sequenced to high depth and with sufficiently long reads (i.e., 150 bp), generally has higher power to detect cell type-driven biases in spatial data. As our knowledge of spatial and even cell type-specific ASE is still in its infancy, it will be an important goal of future studies to query spatial ASE in other contexts to fully understand its potential impact on healthy and diseased states.

## Methods

### Fitting the spASE model

Here, we detail the approach to fitting the spASE model. We denote the counts from the maternal allele for spot *i* and gene *j* with $Y_{i,j}$. We assume the following model:

2 $$\begin{aligned} Y_{i,j}{} & {} \sim \text {Beta-Binomial}(p_{i,j}, N_{i,j}, \phi _j) \nonumber \\ p_{i,j}{} & {} = \sum \limits _{k=1}^K \alpha _{i,j,k} \, \text {expit} \left( \beta _{0,k,j} + \sum \limits _{\ell =1}^L \beta _{\ell ,k,j}\gamma _{i,\ell }\right) , \end{aligned}$$

where $p_{i,j}$ is the mean probability that a transcript from gene *j* is from the maternal allele; $N_{i,j}$ is the total read count, summing both alleles; $\phi _j$ is a gene-specific overdispersion term ranging from 0 to 1 that accounts for biological and technical variability not explained by binomial sampling; $\alpha _{i,j,k}$ are cell type-specific weights; and the $\gamma _{i,\ell }$ represent any potential user-defined covariates of interest, such as categorical regions or a smoothing spline basis function evaluated at continuous spatial coordinates (see below). Note that this parameterization of the Beta distribution in terms of mean and overdispersion corresponds to a Beta($a_{i,j},b_{i,j}$) distribution where $a_{i,j}=p_{i,j}*(1-\phi _j)/\phi _j$ and $b_{i,j}=(1-p_{i,j})*(1-\phi _{j})/\phi _j$.

The proportion of gene *j* on spot *i* belonging to cell type *k*, $\alpha _{i,j,k}$, is pre-computed as:

3 $$\begin{aligned} \alpha _{i,j,k} = \frac{w_{i,k}\mu _{i,j,k}}{\sum \nolimits _k w_{i,k}\mu _{i,j,k}}. \end{aligned}$$

Note that $w_{i,k}$ are the cell type weights (previously called $\beta$) from RCTD, while $\mu _{i,j,k}$ are the cell type-specific gene expression rates from C-SIDE. This can be justified by noting that $\lambda _{i,j}$, the expected maternal proportion at spot *i*, gene *j* satisfies:

4 $$\begin{aligned} \lambda _{i,j}{} & {} = P(m \mid \text {spot i, gene j}) = \sum \limits _k P(m, k \mid \text {spot i, gene j})= \sum \limits _k P(k \mid i,j)P(m \mid i,j,k)\nonumber \\{} & {} = \sum \limits _k \frac{P(k\mid i)P(j\mid k,i)}{P(j \mid i)}\lambda _{i,j,k} = \sum \limits _k \frac{w_{i,k}\mu _{i,j,k}}{\sum \limits _k w_{i,k}\mu _{i,j,k}}\lambda _{i,j,k} = \sum \limits _{k}\alpha _{i,j,k}\lambda _{i,j,k}, \end{aligned}$$

as above.

We provide an algorithm for computing the maximum likelihood estimator of $\beta$, presented in Additional file 1: Supplemental Notes. Our likelihood optimization algorithm is a second-order, trust-region [52] based optimization (Additional file 1: Supplemental Notes) [53]. In brief, we iteratively solve quadratic approximations of the log-likelihood, adaptively constraining the maximum parameter change at each step. Critically, the likelihood is independent for each gene *j* (and sample *g*), so separate genes are run in parallel in which case there are $K\times (L+1)$ parameters per gene and sample.

#### Non-parametric mode

In non-parametric mode, we implement the spline basis functions *B* using the thin plate spline basis [40]. To construct the thin plate spline basis functions evaluated at coordinates $(x_1,x_2)$, we use the following command in R [54]: smoothCon(s(x1,x2,k,fx=T,bs=‘tp’)) for specified degrees of freedom *k* in the package mgcv [55]. For both Slide-seq and Visium data, we experimented with multiple choices of *k*, and selected $k=5$ for testing significant spatial patterns (Tables 1 and 2). We used $k=15$ to provide higher resolution visualizations for genes with a significant spatial pattern (Figs. 3 and 4, and Additional file 1: Figs. S12-S17; see Additional file 1: Supplemental Notes for additional information on the intuition of adjusting *k*).

### Hypothesis testing

The form given in (2) allows for flexibility in choice of covariates $\gamma _{i,\ell }$ depending on the biological hypothesis of interest. Below, we detail the procedure for the categories given in Tables 1 and 2 for each gene *j*.

1. Overall maternal/paternal bias―we assume $\text {logit}(p_{j}) = \beta _{0,j}$, i.e., the mean maternal probability does not change based on cell type or spatial location. We test the significance of the hypothesis $\beta _{0,j} \ne 0$ and require $p_{j}>0.6$ to call a maternal bias and $p_j < 0.4$ to call a paternal bias.
2. Within cell type maternal/paternal bias―we assume $p_{i,j} = \sum \nolimits _{k=1}^K \alpha _{i,j,k} \text {expit}(\beta _{0,k,j})$, and test for the significance of the intercepts $\beta _{0,k,j}$.
3. Overall spatial pattern―we assume $\text {logit}(p_{i,j}) = \beta _{0,j} + \sum \nolimits _{\ell =1}^L B_{i,\ell }\beta _{\ell ,j}$, where $B_{i,\ell }$ are degrees of freedom *L* thin plate spline basis functions evaluated at spots *i*. We compute a likelihood ratio test for the significance of the additional covariates in this model over a baseline intercept model from 1.
4. Within cell type spatial pattern―we assume $p_{i,j} = \sum \nolimits _{k=1}^K \alpha _{i,j,k} \text {expit}(\beta _{0,j} + \sum \nolimits _{\ell =1}^L B_{i,\ell }\beta _{\ell ,j})$ and test for significance of any of the smoothing spline coefficients $\beta _{\ell ,k,j}$.

For all procedures, we employ false discovery rate correction using the Benjamini-Hochberg procedure [56] and use a threshold of $q < 0.01$.

### CAST/EiJ x 129S1/SvImJ F1 mice

We obtained female CAST/EiJ x 129S1/SvImJ (CASTx129) mice from Jackson laboratories (Additional file 1: Fig. S1a). The CASTx129 cross contains $\sim$23 million SNPs or approximately 1 SNP for every $\sim$110 bp [57, 58]. This SNP density is approximately tenfold the SNP density in human cells and thus provides high resolution to interrogate ASE. We also characterized the diversity in the pooled CASTx129 transcriptome (see below) used for alignment. Approximately 40% of the 115,125 total transcripts contained an insertion or deletion when comparing the two strain-specific versions of each transcript. The SNP density was also weakly correlated with the number of counts observed per gene, particularly for transcripts without an indel (Additional file 1: Fig. S1b). The SNP density was variable for different transcripts within a gene, but in general, the primary transcript tended to have a higher density of SNPs, as was the case for *Xist* and *Ptgds* (Additional file 1: Fig. S1c,d). Approximately 100k total SNPs reside in the transcripts for genes measured in this study with non-zero counts.

### Library processing

#### Slide-seq

Slide-seqV2 was performed as described previously [21, 22] on 10-µm-thick coronal slices of the hippocampus from three mice (mice 1–3) and the cerebellum of one mouse (mouse 3). For mice 1 and 3, two serial sections were sequenced and reads were aggregated downstream, while for mouse 2, only one section was sequenced. Slide-seq libraries were processed with the slideseq-tools pipeline (https://github.com/MacoskoLab/slideseq-tools) and then re-aligned to a custom transcriptome (see below). For longer read libraries (mouse 3 Slide-seq), we used Atropos [59] to trim adapter sequences and low quality bases.

#### Visium

10x Genomics Visium was performed on two mice (Mice 4,5) on 10-µm-thick coronal slices of the cerebellum. Visium libraries were processed with the 10x Genomics Space Ranger 1.1.0 pipeline and then re-aligned to a custom transcriptome (see below).

### Alignment

#### Read alignment

We generated a pooled CASTx129 transcriptome using the command create-hybrid from the EMASE [60] software on the CAST and 129 transcript fasta files downloaded from ftp://churchill-lab.jax.org/software/g2gtools/mouse/R84-REL1505/. We then aligned reads to this pooled transcriptome with bowtie2 [61] using the parameters-k 100 -p 16 –very-sensitive. This method of alignment ensures there is no reference bias. The multi-mapping parameter $k=100$ was chosen to report a large amount of multi-maps as bowtie2 randomly reports multi-mapping locations (in order of increasing number of mismatches). We used a custom script (https://github.com/lulizou/spASE/blob/master/scripts/processBowtie2.py) for processing the aligned BAM file [62] to create a gene UMI count matrix only from reads that uniquely aligned to one gene and one allele. We conservatively restricted attention to alignments with 3 or fewer mismatches and only considered alignments that had the fewest number of mismatches for that read. We merged the reads from samples that had two serial sections: mouse 1 hippocampus, mouse 3 hippocampus, and mouse 3 cerebellum.

#### Spatial alignment

We overlaid data from the Slide-seq samples with two the two serial sections (mouse 1, mouse 3) by rotating, shifting, and scaling the slices to overlap according to the location of the hippocampal formation and the granule layer of the cerebellum.

### Cell type assignment

We ran RCTD [23] in doublet mode (default settings: MIN.CHANGE = 0.001, CONFIDENCE_THRESHOLD = 10, DOUBLET_THRESHOLD = 25) for the Slide-seq samples and full mode (default settings: MIN.CHANGE = 0.001) for the Visium samples. We used all UMIs, including those aligned to genes but not aligned to an allele. We used previously published hippocampus [63] and cerebellum [64] scRNA-seq datasets as references. Cell type weights were normalized to sum to 1 for each spot for downstream analysis.

### Cell type-specific differential expression

We ran C-SIDE [43] as previously described in non-parametric mode, filtering to gene and cell type combinations with at least 128 spots with non-zero counts. We selected degrees of freedom to match the degrees of freedom used to fit the non-parametric spASE functions.

### Simulations

To evaluate the ability of spASE to estimate ASE in the presence of cell type mixtures, we simulated ground truth 2D maternal allele probability functions (Fig. 2). Starting with the mouse cerebellum data collected using Visium and Slide-seqV2, we first used RCTD and C-SIDE to compute cell type weights and cell type-specific spatial DE estimates, respectively, for each gene. We focused on the fibroblast, granule, MLI2, and oligodendrocyte cell types as they had the highest sample size in both the Visium and Slide-seqV2 data. Then, for each gene in a given cell type, we generated a 2D maternal allele probability function using random linear combinations of basis functions with five degrees of freedom. In addition to varying which cell type and which gene we focused on, we also varied the amount of ground truth gene-specific overdispersion ($\phi _j$ above) and the total UMI count for that gene. Simulation performance was evaluated using the correlation *r* between the ground truth maternal probability function and the estimated function, as well as the root-mean-squared error (RMSE) of the estimated coefficients (Additional file 1: Fig. S9).

### Mouse hippocampus scATAC-seq, *cis*-regulatory elements, and TFBS motifs

We analyzed previously published mouse hippocampus sci-ATAC-seq count matrices (GSE118987) [65]. The mice used in the study were the wild-type C57/B6 strain, and the data were aligned to the mm10 genome. We extracted the called peak annotations and counts using the command scitools split. To quantify accessibility of peaks, we computed the average count within cell types. We searched for known TFBS motifs within peaks with the command matchMotifs from the R package motifmatchr [66] using all motifs for *Mus musculus* in the JASPAR 2020 database [67]. We overlaid annotations of ENCODE cis-regulatory elements (cCREs) [68] for mm10 downloaded from the UCSC Table Browser [69] at the *Ptgds* locus and visualized the annotations using IGV [70].

### Mouse strain SNP data

We obtained gene-specific SNP annotations for 129S1/SvImJ and CAST/EiJ with respect to the mm10 reference using REL1505 of the Mouse Genomes Project (https://www.sanger.ac.uk/sanger/Mouse_SnpViewer/rel-1505) [71, 72].

### Mouse scRNA-seq data

We downloaded mouse scRNA-seq data from the Allen Brain Map [73], filtered to cell types annotated that were present in our mouse hippocampus data set, and visualized the expression of *Mtf1* (Additional file 1: Fig. S12).

### Supplementary Information

Additional file 1: Table S1 Visium and Slide-seq (Puck) data generated in this study. Fig. S1 Schematic and comparison of the F1 hybrid mice and pooled transcriptome used in this study. Fig. S2 Distributions of total read counts and total allele-resolved read counts for each sample. Fig. S3 Cell type maps from Slide-seq data gathered on three mice from the hippocampus. Fig. S4 Cell type maps from Slide-seq data gathered on one mouse from the cerebellum. Fig. S5 Histology and cell type map for Visium on the cerebellum from Mouse 4. Fig. S6 Histology and cell type map for Visium on the mixture (cerebellum and nearby region) from Mouse 5. Fig. S7 Allele-resolved Visium and Slide-seq generated from two F1 hybrid (CAST x 129) mouse cerebellums. Fig. S8 Distributions of maternal count proportion by spot (for Slide-seq and Visium) or by cell (for external Smart-seq3) data, stratified by the total (maternal $+$ paternal) UMI count, per gene and per spot or cell. Fig. S9 Root-mean-squared error for estimated coefficients for ground truth simulations vs. total UMI generated for a gene in that simulation. Fig. S10 C-SIDE estimates and variances for all data sets analyzed in this study. Fig. S11 Sample comparisons for overall estimated maternal proportion ($\hat{p}$) by gene. Fig. S12 Cell type-specific open chromatin and motif analysis for the gene *Ptgds* in oligodendrocytes as a potential explanation for the paternal-specific expression observed in the mouse hippocampus. Fig. S13 Fitted 2D smooth functions for X-chromosome genes in the mouse 1 hippocampus. Fig. S14 Fitted 2D smooth functions for X-chromosome genes in the mouse 2 hippocampus. Fig. S15 Fitted 2D smooth functions for X-chromosome genes in the mouse 3 hippocampus. Fig. S16 Fitted 2D smooth functions for X-chromosome genes in the mouse 3 cerebellum. Fig. S17 Fitted 2D smooth functions for X-chromosome genes in the mouse 4 cerebellum (Visium). Confidence intervals for dotted line cross-sections are shown for *Tceal3*, *Tceal5*, and *Tceal6*. Fig. S18 Visium merged X-chromosome fits. Fig. S19 Estimated maternal probability cross-sections along the $x_2$ direction for X-chromosome genes that were previously shown to escape XCI.

Additional file 2: Table S2 List of genes detected with an overall maternal or paternal bias at $q < 0.01$ for each sample.

Additional file 3: Table S3 List of genes detected as having a within cell type maternal or paternal bias at $q < 0.01$ for each sample.

Additional file 4: Table S4 List of genes detected as having an overall spatial pattern at $q < 0.01$ for each sample.

Additional file 5: Table S5 List of genes detected as having an within cell type spatial pattern at $q < 0.01$ for each sample.

Additional file 6: Review history.

## Data Availability

Raw and processed Slide-seq and Visium data generated in this study is available at GEO (https://www.ncbi.nlm.nih.gov/geo/query/acc.cgi?acc=GSE268519) [74] as well as the Broad Single Cell Portal (https://singlecell.broadinstitute.org/single_cell/study/SCP1692). Single-cell ATAC-seq from the mouse hippocampus was downloaded from GSE118987 (https://www.ncbi.nlm.nih.gov/geo/query/acc.cgi?acc=GSE118987) [65, 75]. Mouse SNP annotations were obtained from REL1505 of the Mouse Genomes Project (https://www.sanger.ac.uk/sanger/Mouse_SnpViewer/rel-1505) [71, 72]. Mouse single-cell RNA-seq (10X Genomics) for the gene *Mtf1* was downloaded from the Allen Brain Map (https://portal.brain-map.org/atlases-and-data/rnaseq) [73]. Our software is freely available under and implemented as an R package on GitHub located at https://github.com/lulizou/spase [76]. The code to reproduce the results of this manuscript is located at https://github.com/lulizou/spASE/tree/master#reproducibility [76]. All scripts and analyses are also deposited in Zenodo at 10.5281/zenodo.11239156 [77].
