# Supplementary material for: Detection of allele-specific expression in spatial transcriptomics with spASE
Source: Genome Biol. 2024 Jul 8;25:180. doi: 10.1186/s13059-024-03317-4 (PMC11229351; doi:10.1186/s13059-024-03317-4)
Supplement: Supplementary file 1 — Additional file 1: Table S1 Visium and Slide-seq (Puck) data generated in this study. Fig. S1 Schematic and comparison of the F1 hybrid mice and pooled transcriptome used in this study. Fig. S2 Distributions of total read counts and total allele-resolved read counts for each sample. Fig. S3 Cell type maps from Slide-seq data gathered on three mice from the hippocampus. Fig. S4 Cell type maps from Slide-seq data gathered on one mouse from the cerebellum. Fig. S5 Histology and cell type map for Visium on the cerebellum from Mouse 4. Fig. S6 Histology and cell type map for Visium on the mixture (cerebellum and nearby region) from Mouse 5. Fig. S7 Allele-resolved Visium and Slide-seq generated from two F1 hybrid (CAST x 129) mouse cerebellums. Fig. S8 Distributions of maternal count proportion by spot (for Slide-seq and Visium) or by cell (for external Smart-seq3) data, stratified by the total (maternal $+$+ paternal) UMI count, per gene and per spot or cell. Fig. S9 Root-mean-squared error for estimated coefficients for ground truth simulations vs. total UMI generated for a gene in that simulation. Fig. S10 C-SIDE estimates and variances for all data sets analyzed in this study. Fig. S11 Sample comparisons for overall estimated maternal proportion ($\hat{p}$p^) by gene. Fig. S12 Cell type-specific open chromatin and motif analysis for the gene Ptgds in oligodendrocytes as a potential explanation for the paternal-specific expression observed in the mouse hippocampus. Fig. S13 Fitted 2D smoot [file 13059_2024_3317_MOESM1_ESM.pdf]

Table S1: Visium and Slide-seq (Puck) data generated in this study. All samples are from female, genetically identical F1 hybrid mice (CAST x 129). "Allele-resolved" is the average number of reads per spot that were able to be uniquely assigned to one allele. Note that these are the number of counts and genes before downstream filtering. *Also note that although the libraries were 250bp, the quality towards the end of the reads was highly variable, and we trimmed low quality bases; thus the actual read lengths used for alignment were on average smaller than 250bp. **This sample contained part of the cerebellum but otherwise a different brain region; therefore we did not include it in our cerebellum-specific analysis, but we did include it in our overall X-chromosome analysis (Figure 4l, Supplemental Figure S6).

Mouse	Tissue	Platform	Read length	N spots	N genes	Avg. reads/spot	Allele-resolved
1	Hippo	Slide-seq	160	26,429	22,049	459	207
2	Hippo	Slide-seq	56	13,680	23,541	623	145
3	Hippo	Slide-seq	250*	78,806	29,411	552	218
3	Cere	Slide-seq	250*	60,942	30,658	622	240
4	Cere	Visium	91	4,315	19,860	2,219	834
5	Mix**	Visium	91	4,150	20,095	2,143	853

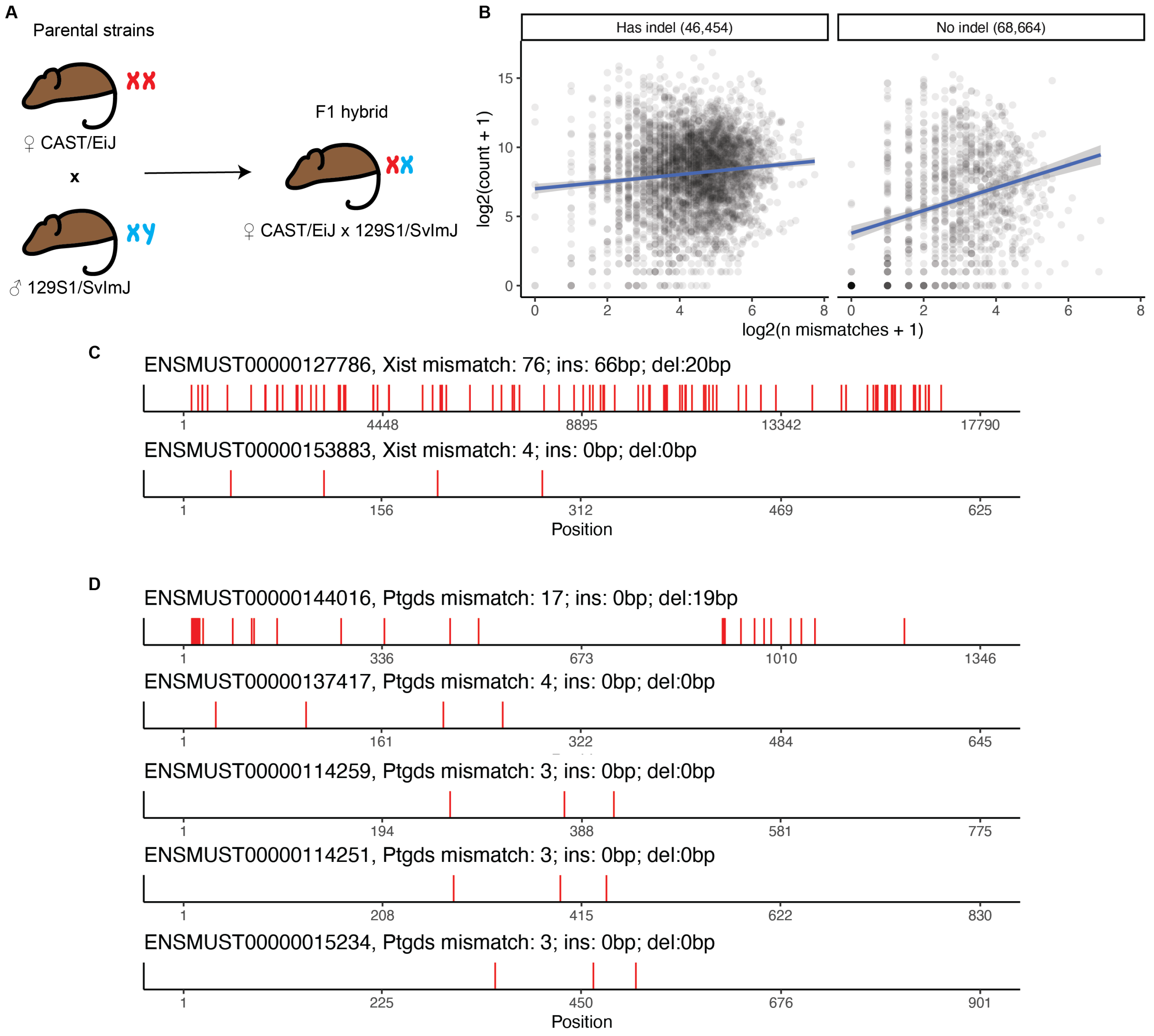

Fig. S1: Schematic and comparison of the F1 hybrid mice and pooled transcriptome used in this study. **(a)** Schematic of the CASTx129 F1 hybrid cross used. All samples were collected from female F1 hybrid mice. **(b)** Scatterplot of genes showing the total number of UMIs measured vs. the number of mismatches in that gene between the two strains, stratified by whether or not there was an indel or not. We selected the transcript with the maximum number of mismatches for each gene. The total count of UMIs is determined by the UMI counts for the gene (i.e. not transcript-level UMIs). Panel titles show the number of transcripts with and without indels. **(c)** The two transcripts for *Xist*, showing one primary, long transcript with 76 mismatches and one secondary transcript. Red lines indicate areas of disagreement (either mismatch or indel) between the two strains. **(d)** Transcripts for *Ptgds*, again with one primary transcript in the top row followed by less common, shorter transcripts.

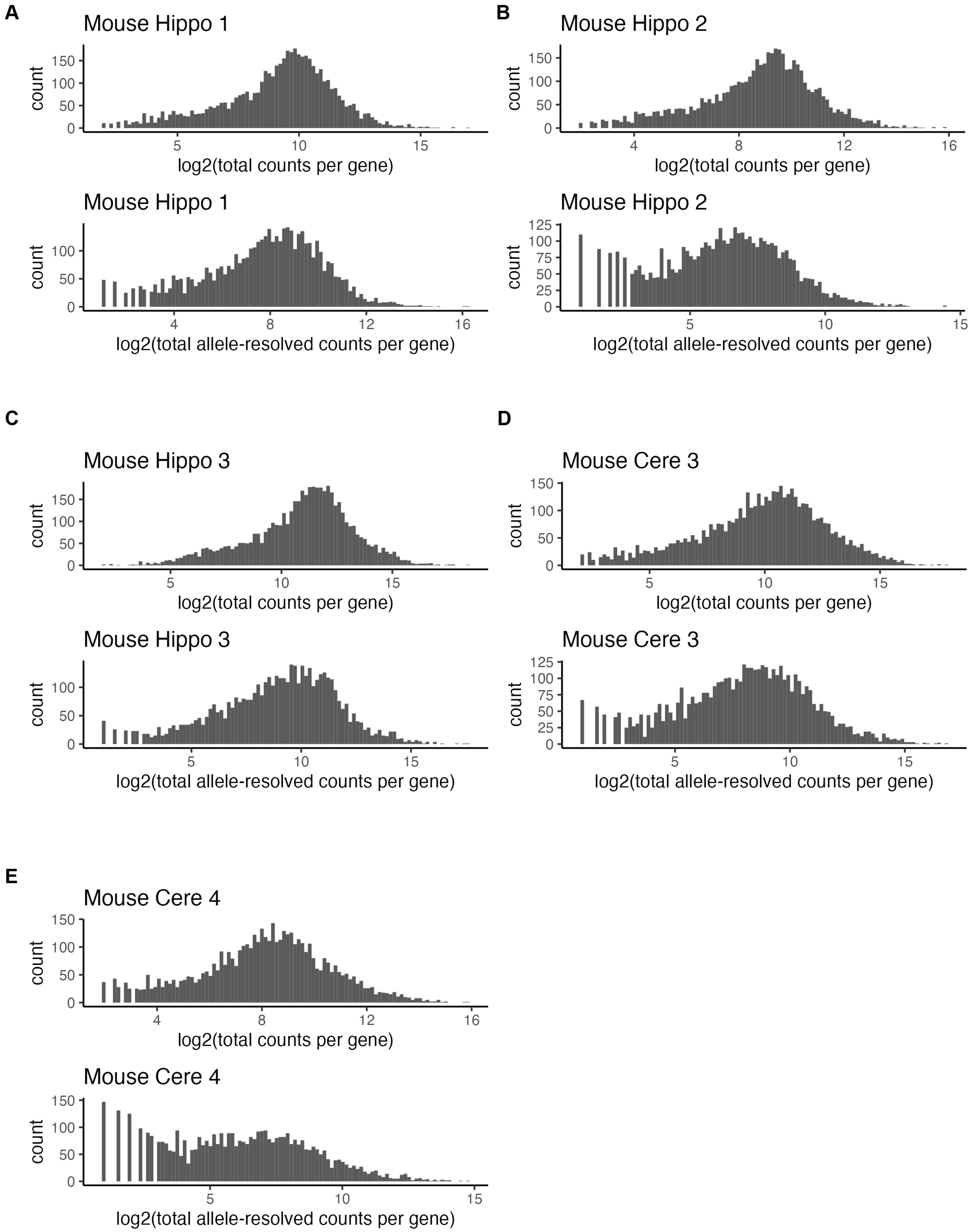

Fig. S2: (a-e) Distributions of total read counts and total allele-resolved read counts for each sample.

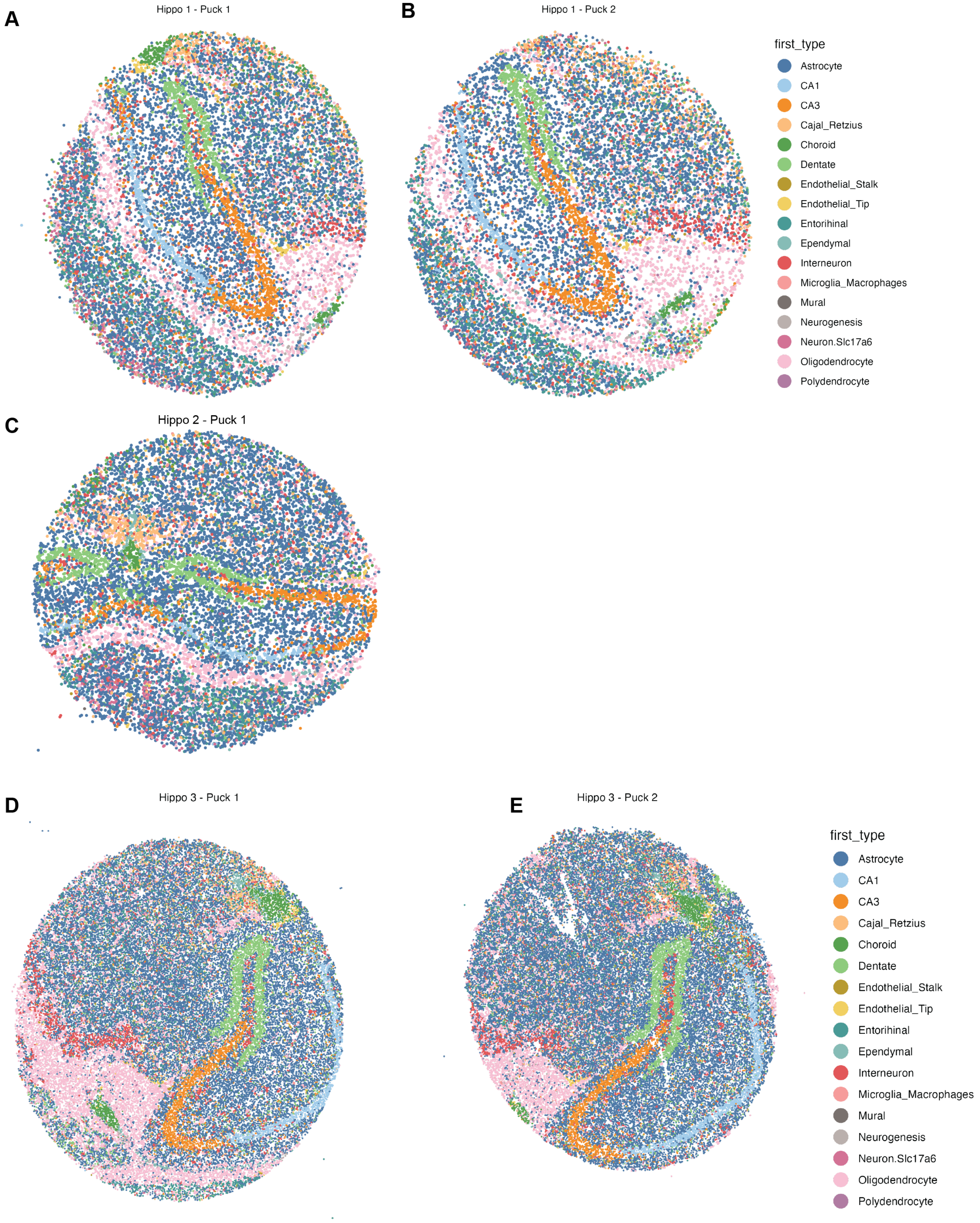

Fig. S3: Cell type maps from Slide-seq data gathered on three mice from the hippocampus. **(a-b)** Pucks generated from mouse 1. **(c)** Puck generated from mouse 2. **(d-e)** Pucks generated from mouse 3.

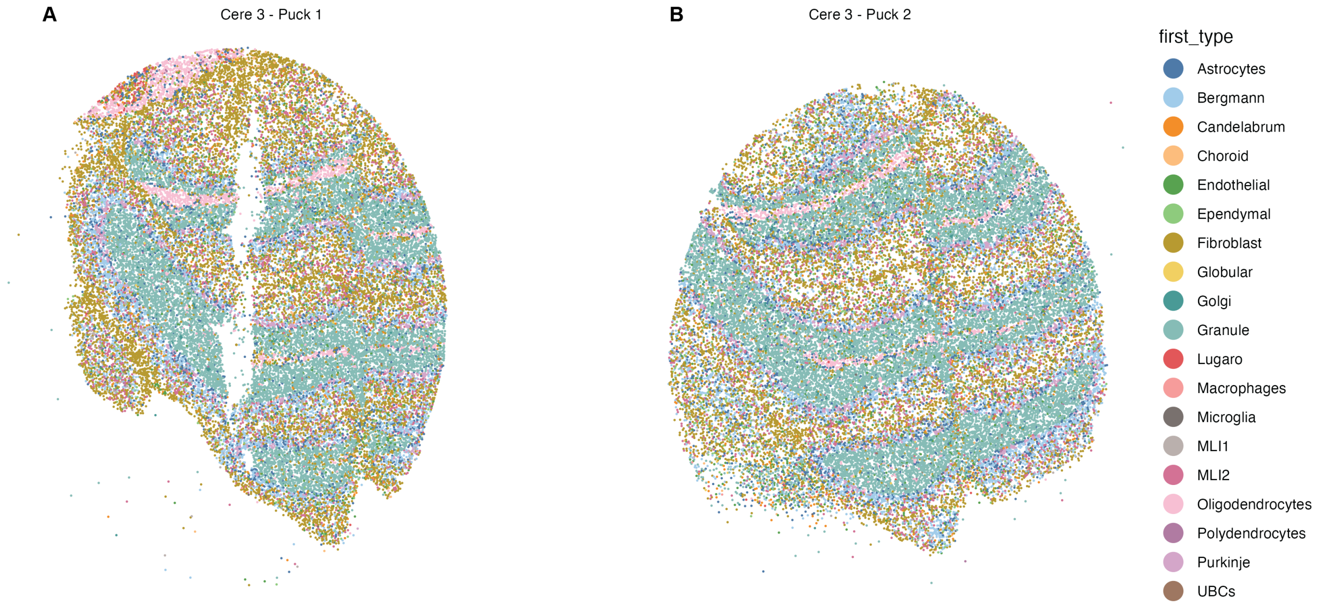

Fig. S4: Cell type maps from Slide-seq data gathered on one mouse from the cerebellum. **(a-b)** Pucks generated from mouse 3.

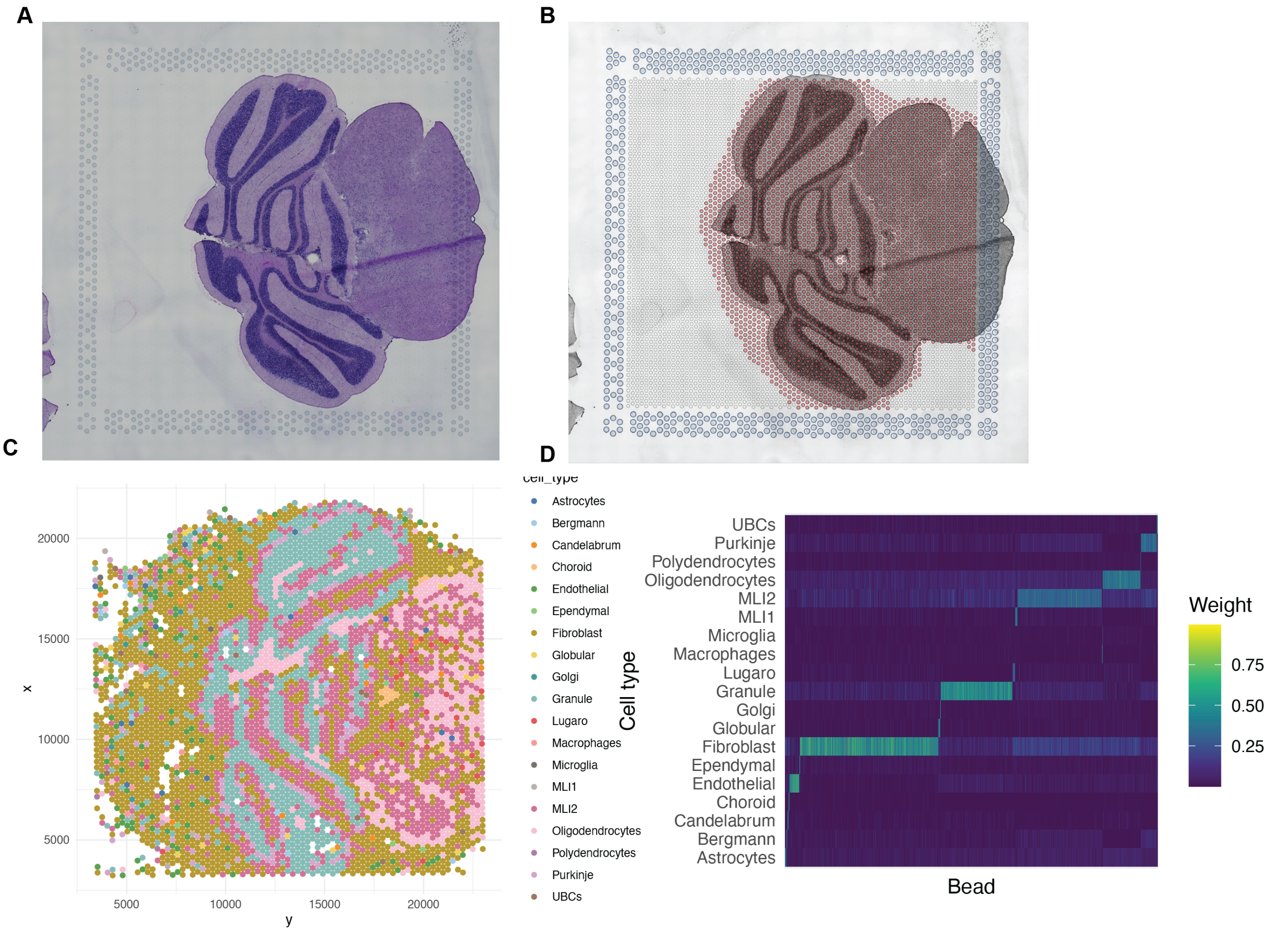

Fig. S5: Histology and cell type map for Visium on the cerebellum from Mouse 4. **(a)** H&E stain of the slice sampled. **(b)** Image showing sampled spots on the slice. **(c)** Cell type classification map from RCTD. **(d)** Cell type weights by spot (bead).

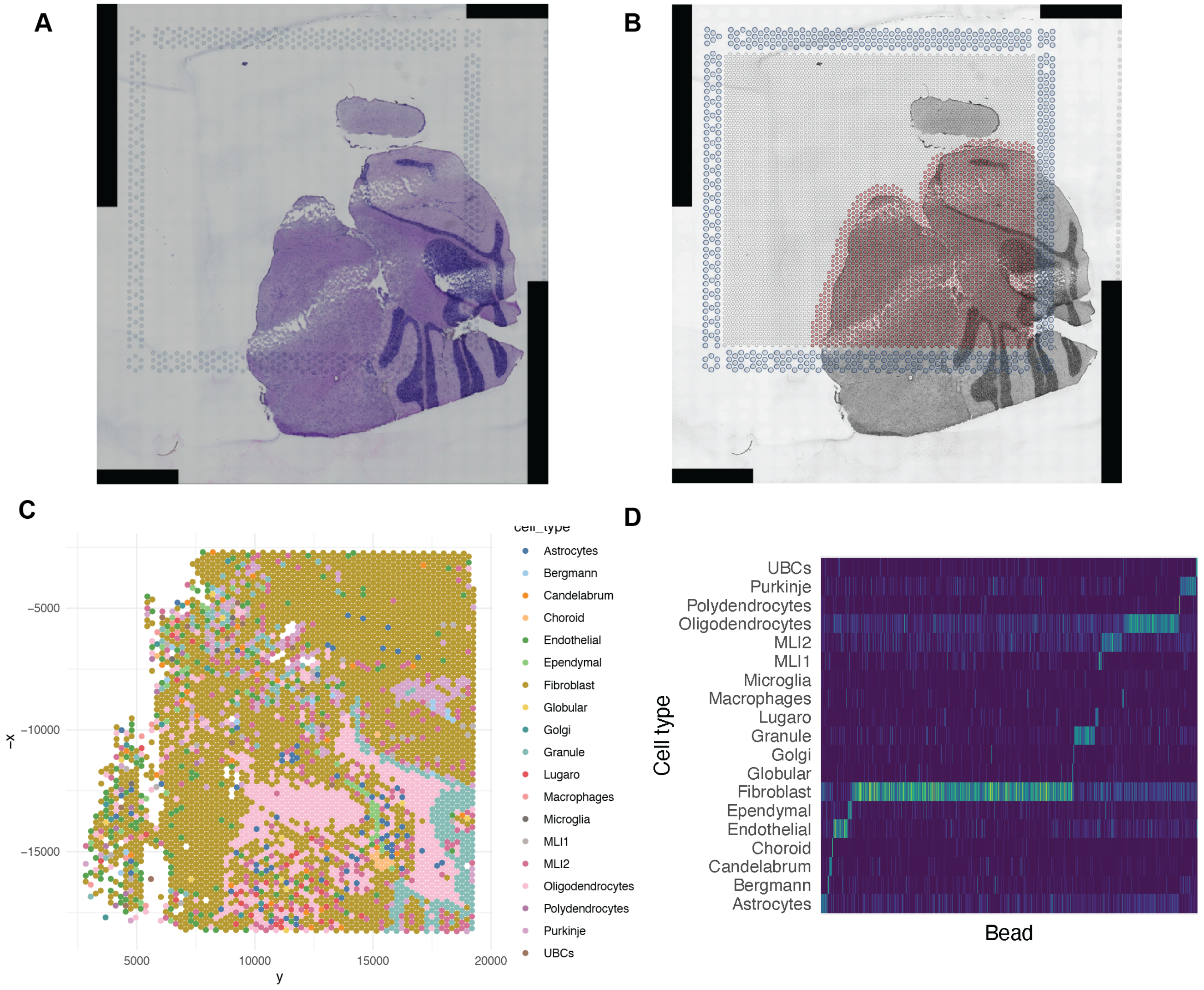

Fig. S6: Histology and cell type map for Visium on the mixture (cerebellum and nearby region) from Mouse 5. **(a)** H&E stain of the slice sampled. **(b)** Image showing sampled spots on the slice. **(c)** Cell type classification map from RCTD. **(d)** Cell type weights by spot (bead).

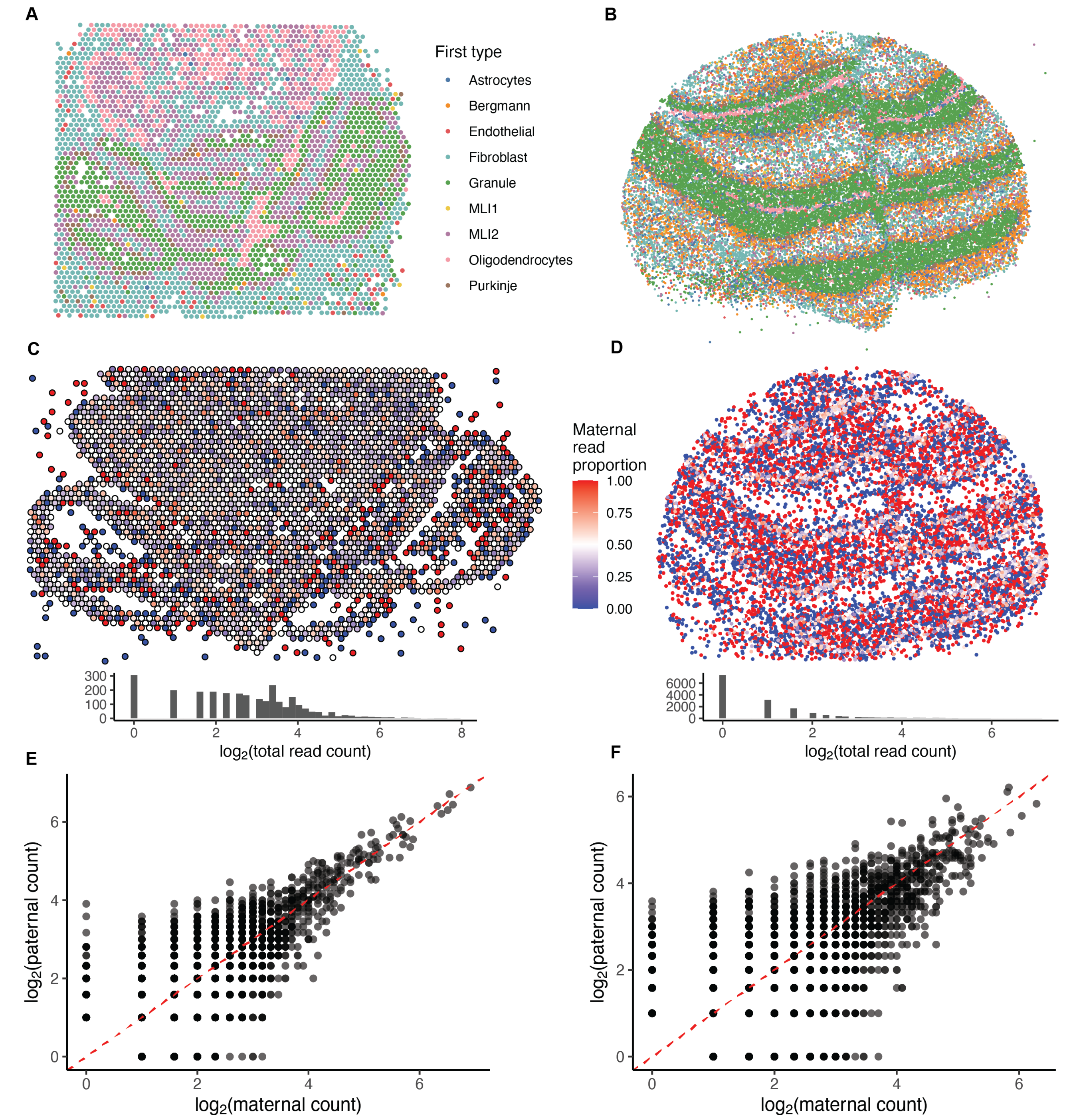

Fig. S7: Allele-resolved Visium (left) and Slide-seq (right) generated from two F1 hybrid (CAST x 129) mouse cerebellums. (a-b) Cell type maps for Visium and Slide-seq data, respectively. Each spot is colored by its primary (first) assigned cell type, but many spots contain mixtures. **(c-d)** Allele-resolved maps for the gene *Aldoc* in Visium and Slide-seq, respectively. Circles indicate measurement locations with non-zero allele-resolved counts for *Aldoc*. Color indicates fraction of total transcripts that were from the maternal allele. Histograms show the distribution of total counts observed at each spot. **(e-f)** Scatterplots showing the maternal and paternal read count for each spot for the gene *Aldoc* for Visium and Slide-seq, respectively.

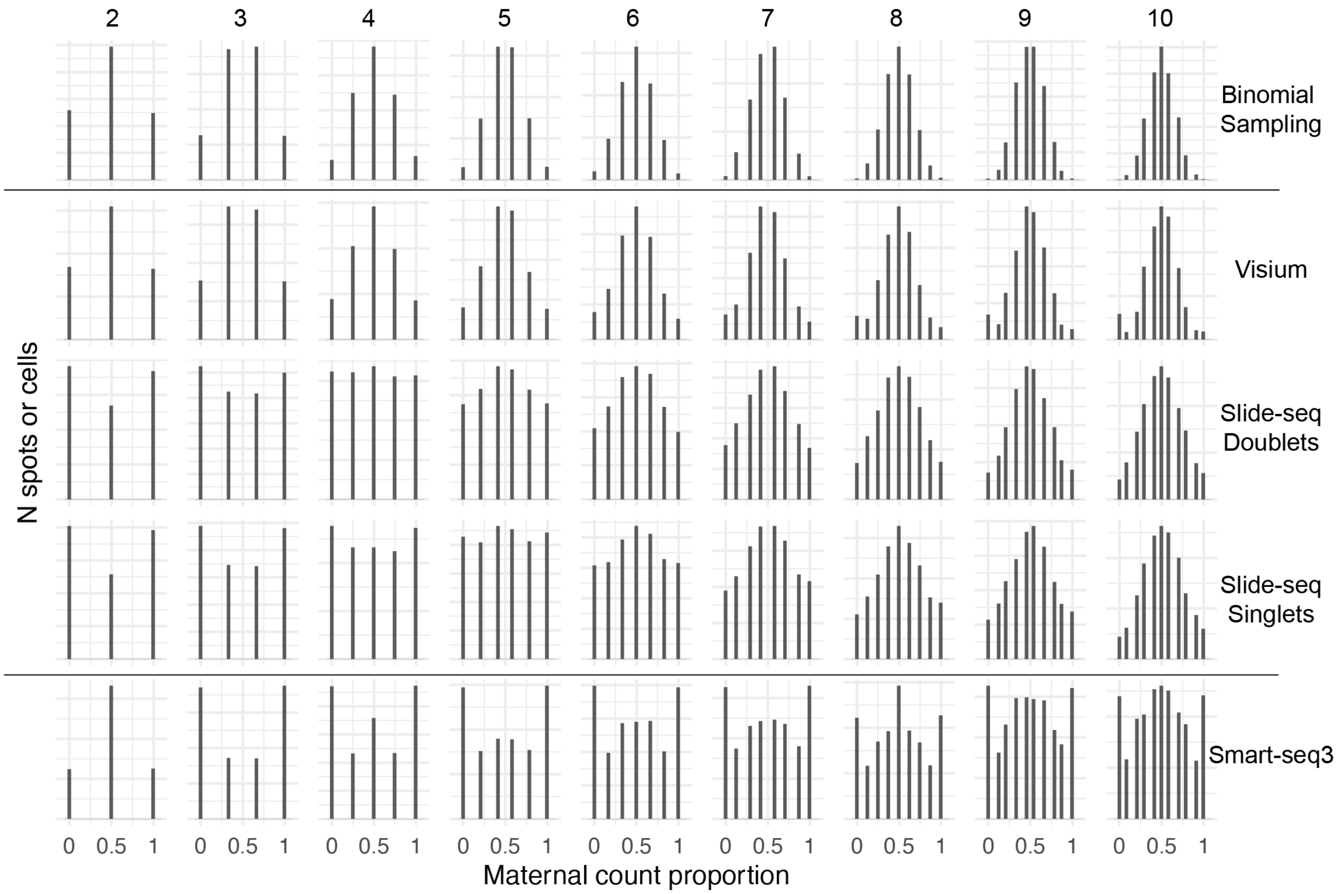

Fig. S8: Distributions of maternal count proportion by spot (for Slide-seq and Visium) or by cell (for external Smart-seq3) data, stratified by the total (maternal + paternal) UMI count, per gene and per spot or cell. Histograms include counts from all spots (or cells) and all genes. For example: column 2 shows the distribution of the maternal count proportion (maternal count / total count) for all spots or cells that had 2 total UMI counts for a given gene; thus, the possible values of the maternal count proportion are only 0/2, 1/2, or 2/2. Spots may be represented more than once in a single panel, since multiple genes could have 2 total UMIs at that spot. Top row shows simulated data of perfect binomial sampling with $p = 0.5$ (no allelic bias).

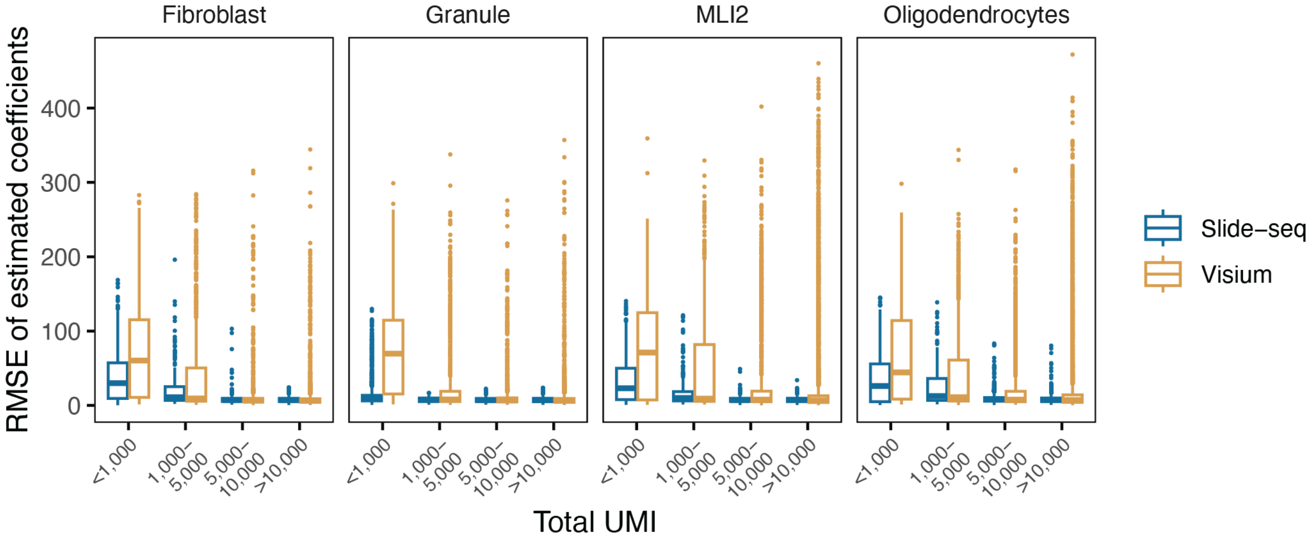

Fig. S9: Root-mean-squared error for estimated coefficients for ground truth simulations vs. total UMI generated for a gene in that simulation. Panels show which cell type contained the cell type-specific ASE effect. Colors indicate the data set used to sample the cell locations and estimate the cell type-specific weights and DE values.

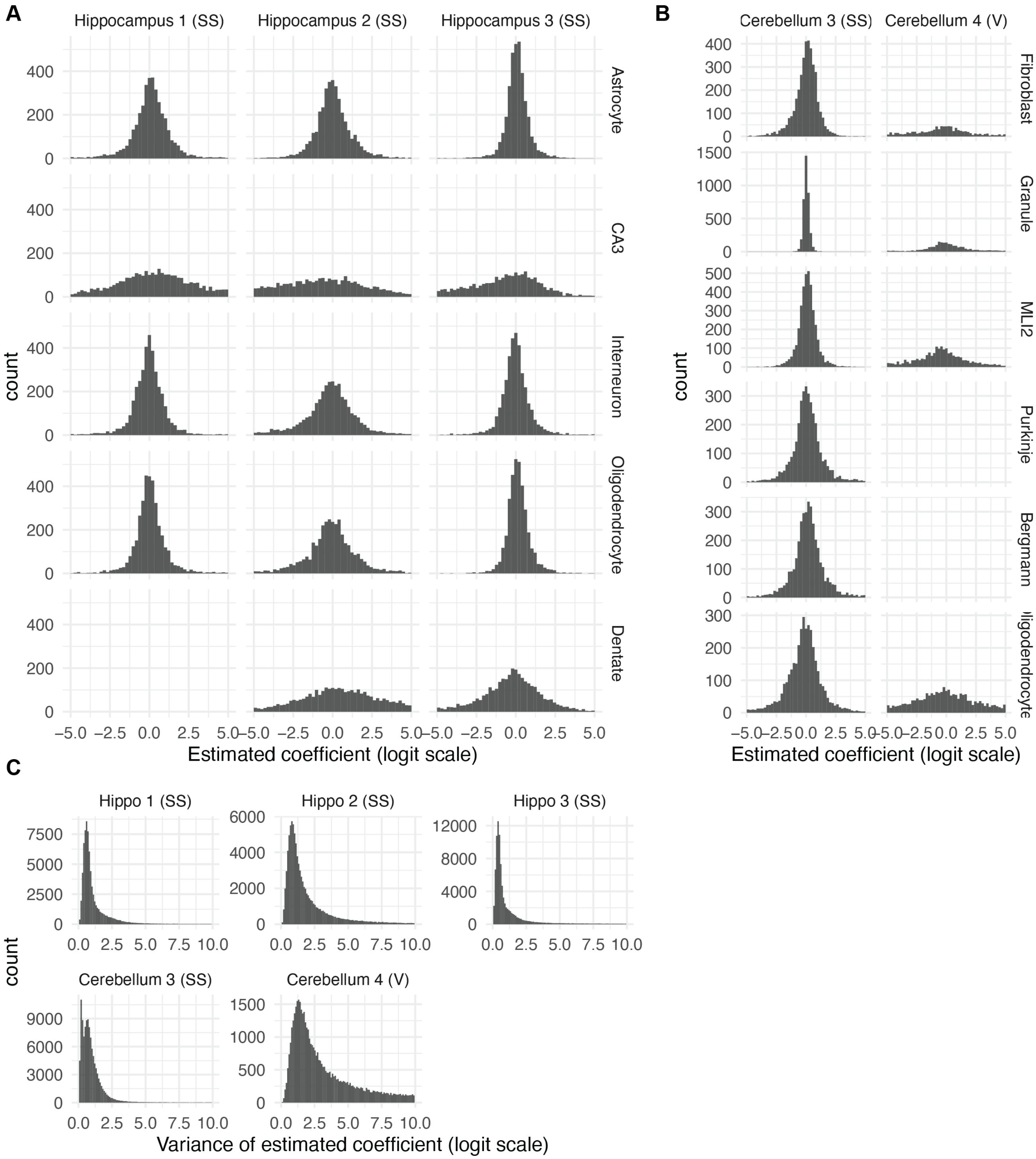

Fig. S10: C-SIDE estimates and variances for all data sets analyzed in this study. **(a)** C-SIDE cell type-specific differential expression coefficient estimates on all hippocampus Slide-seq samples, using non-parametric mode with degrees of freedom 5. Dentate was not estimated in Hippocampus 1 due to lower sample size. **(b)** Same as (a) but for Slide-seq and Visium samples from the cerebellum. Purkinje and Bergmann were not estimated in Visium due to small sample size. **(c)** Histograms of variances of the estimate for each sample.

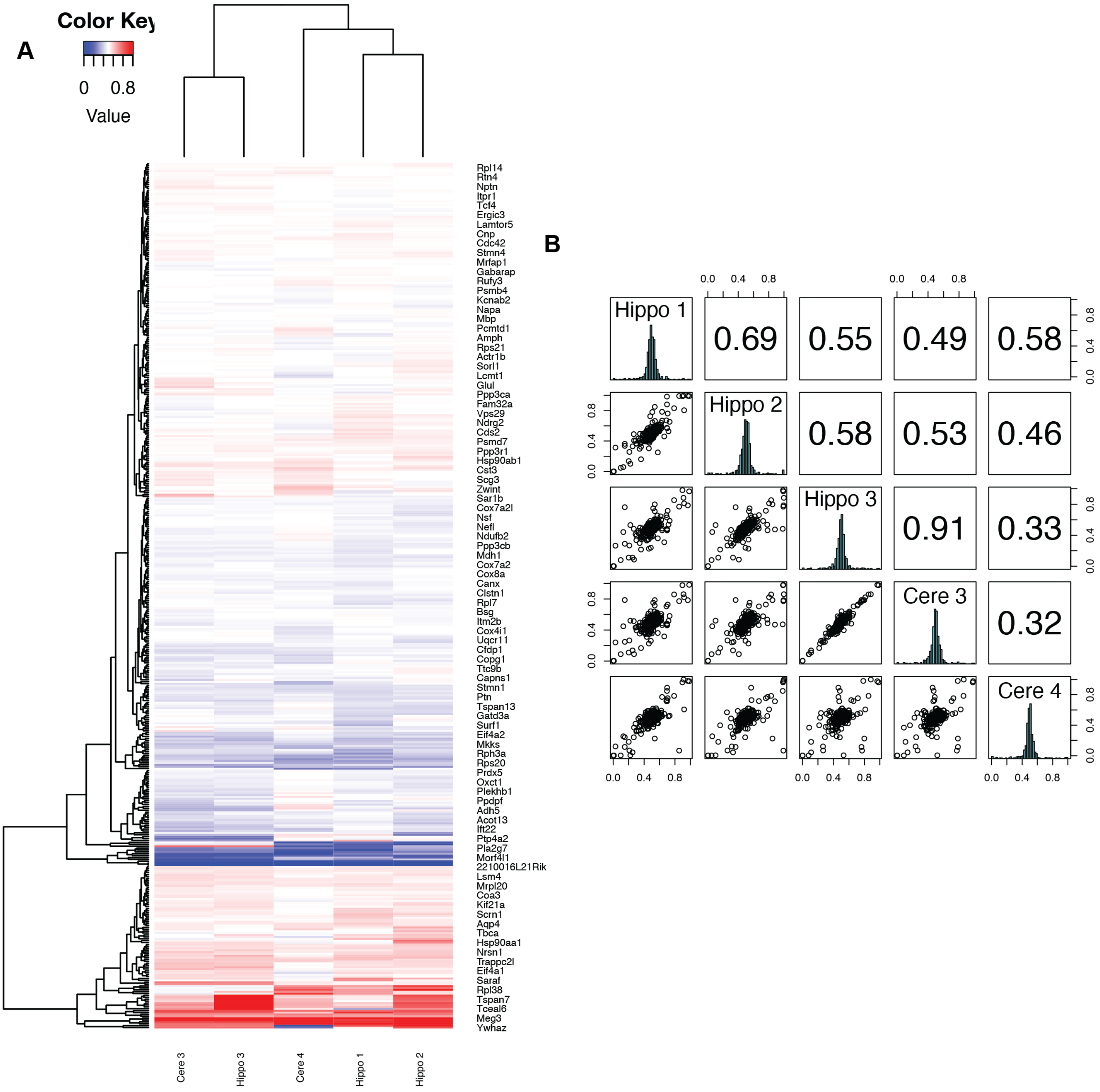

Fig. S11: Sample comparisons for overall estimated maternal proportion ($\hat{p}$) by gene. **(a)** Heatmap showing $\hat{p}$ for each gene in each sample from the beta-binomial fit with no spatial or cell type effect, filtered to genes with at least 500 UMIs present in each sample. Note: row names do not show all genes. For a list of genes and estimates see Supplemental Table **??**. **(b)** Scatterplot and R^2 comparing $\hat{p}$ across samples, again filtered to genes with at least 500 UMIs present in each sample.

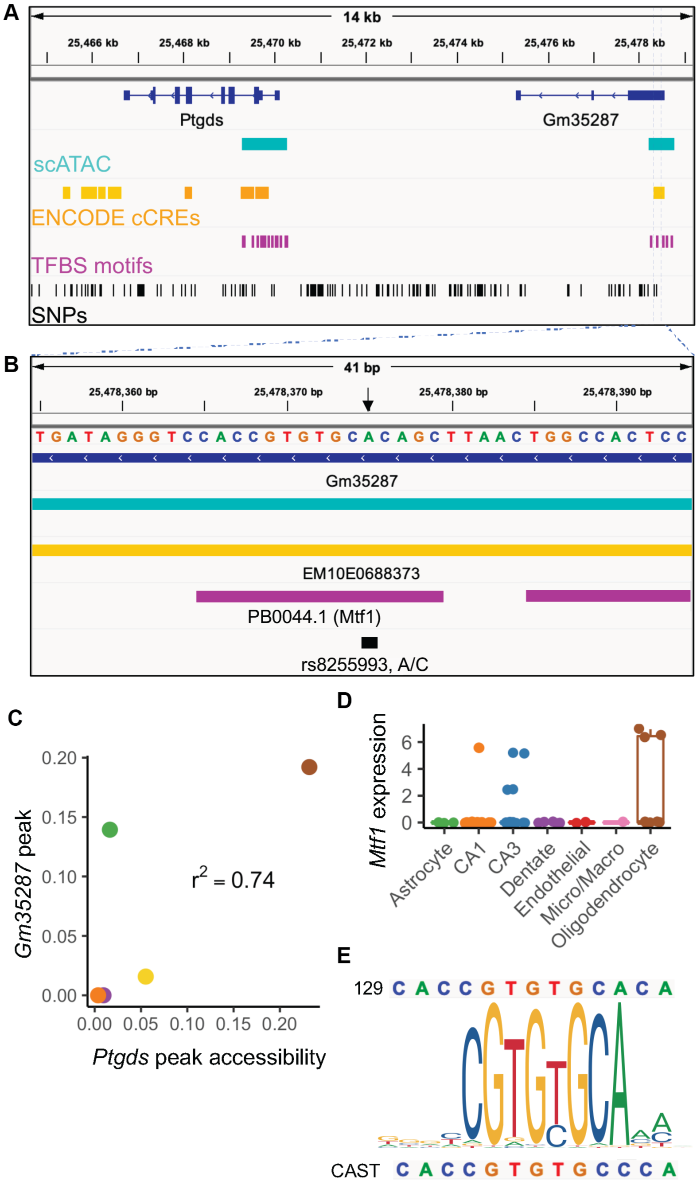

Fig. S12: Cell type-specific open chromatin and motif analysis for the gene *Ptgds* in oligodendrocytes as a potential explanation for the paternal-specific expression observed in the mouse hippocampus from Figure 3. **(a)** IGV view (mm10) of the *Ptgds* locus showing the upstream *Gm35287* locus. Dark blue indicates Refseq gene annotation, cyan indicates peaks called from sci-ATAC-seq data from the mouse hippocampus, yellow denotes cis-regulatory elements (cCREs) from the ENCODE database (lighter yellow indicates distal-TSS enhancer-like signatures, darker yellow indicates proximal-TSS enhancer like signatures), magenta indicates predicted transcription factor binding site motifs within sci-ATAC-seq peaks, black indicates SNP locations for the CAST/EiJ and 129S1/SvmJ strains relative to mm10. **(b)** Zoomed-in genome browser view of the PB0044.1 motif (*Mtf1* gene) located in the peak overlapping *Gm35287*. **(c)** Average sci-ATAC-seq peak accessibility of the *Ptgds* promoter peak and the nearby (∼8kb away) peak in *Gm35287* for the cell types overlapping between the sci-ATAC-seq data set and the Slide-seq data. **(d)** Single-cell RNA-seq expression for *Mtf1* from the Mouse Brain Atlas [73]. Each point represents a cluster that was classified as one cell type. **(e)** Position weight matrix for PB0044.1 (*Mtf1*) with 129 (paternal) and CAST (maternal) reference sequences shown on the top and bottom, respectively. Black arrow points to the SNP position of interest.

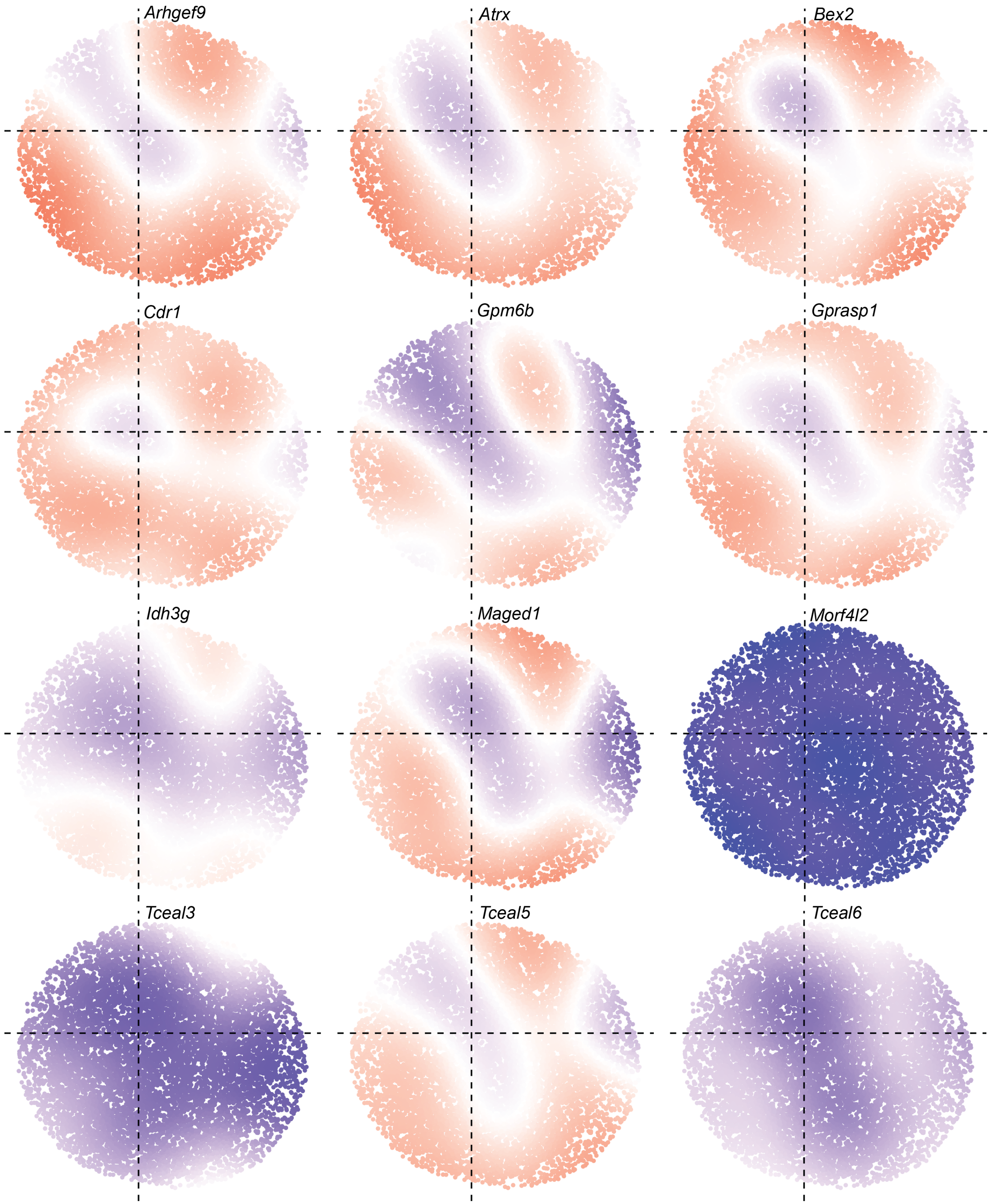

Fig. S13: Fitted 2D smooth functions for X-chromosome genes in the mouse 1 hippocampus.

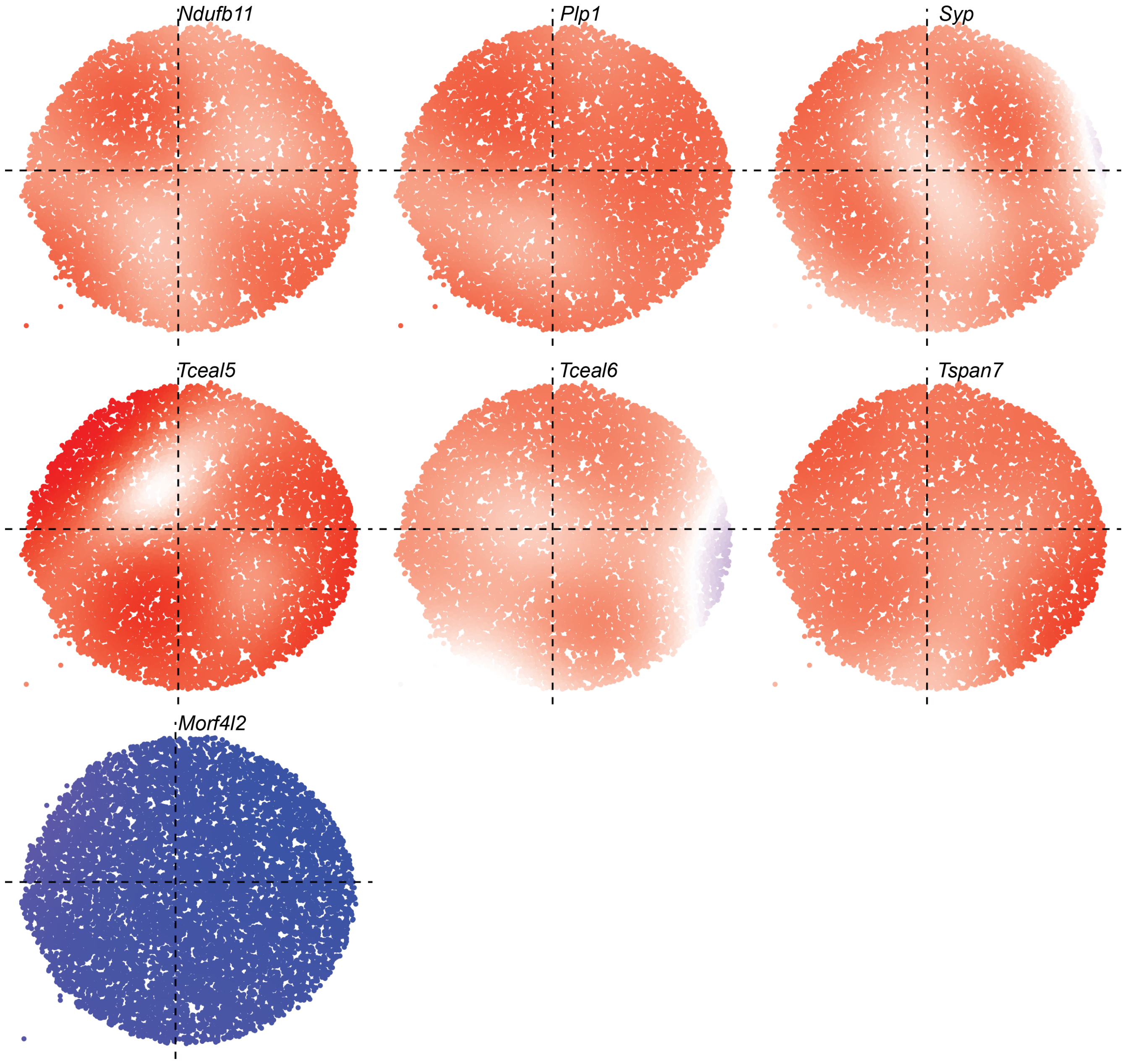

Fig. S14: Fitted 2D smooth functions for X-chromosome genes in the mouse 2 hippocampus.

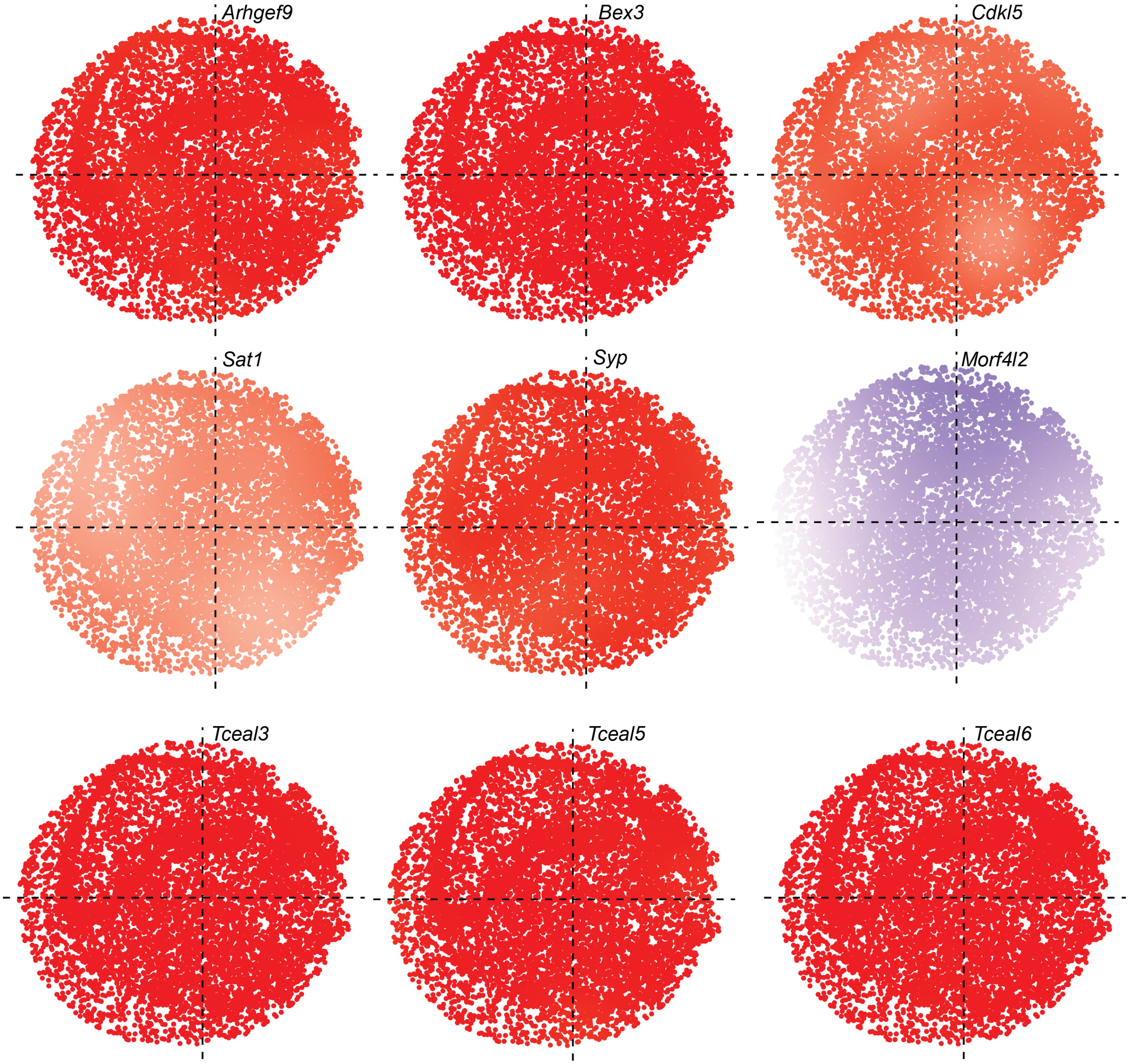

Fig. S15: Fitted 2D smooth functions for X-chromosome genes in the mouse 3 hippocampus.

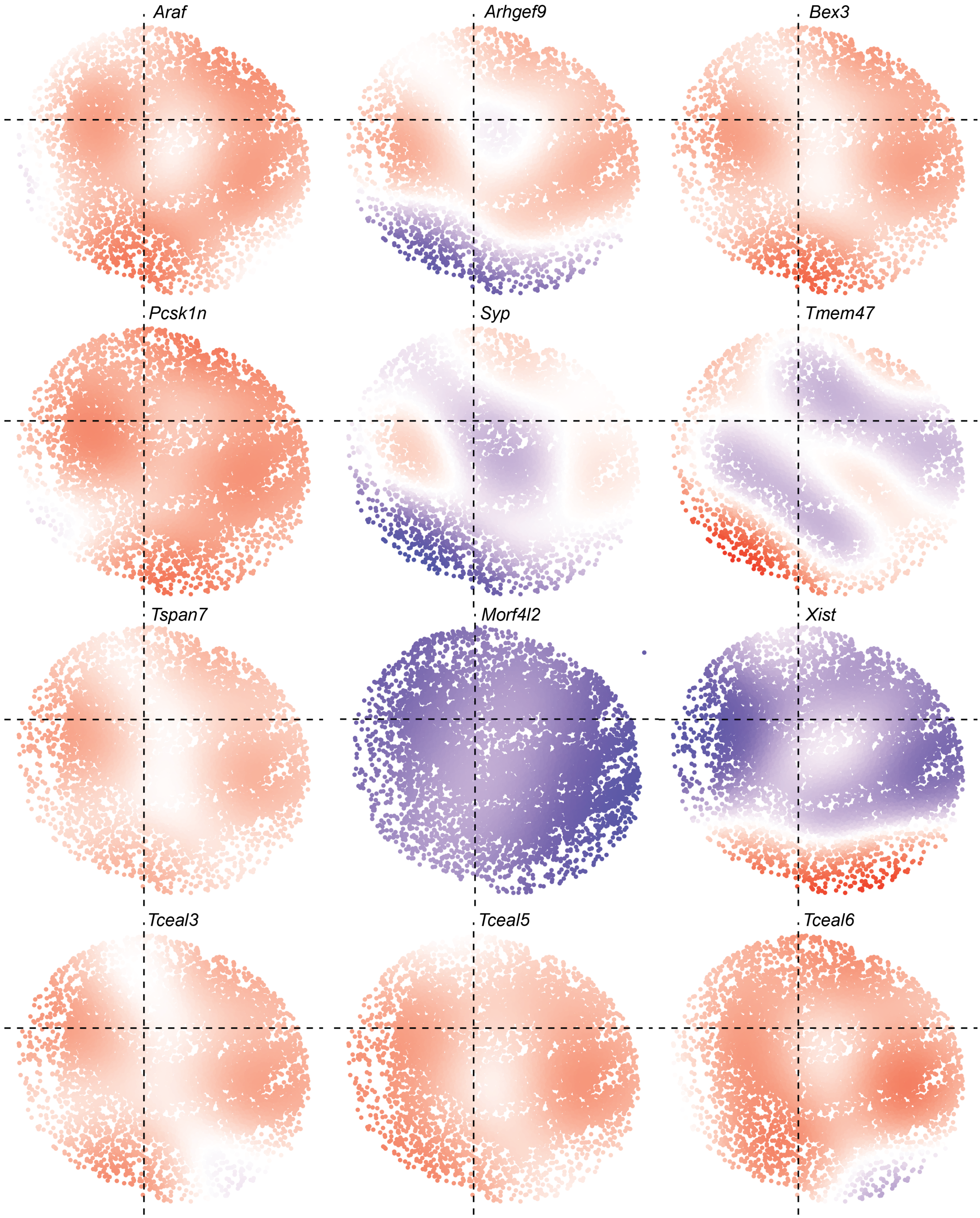

Fig. S16: Fitted 2D smooth functions for X-chromosome genes in the mouse 3 cerebellum.

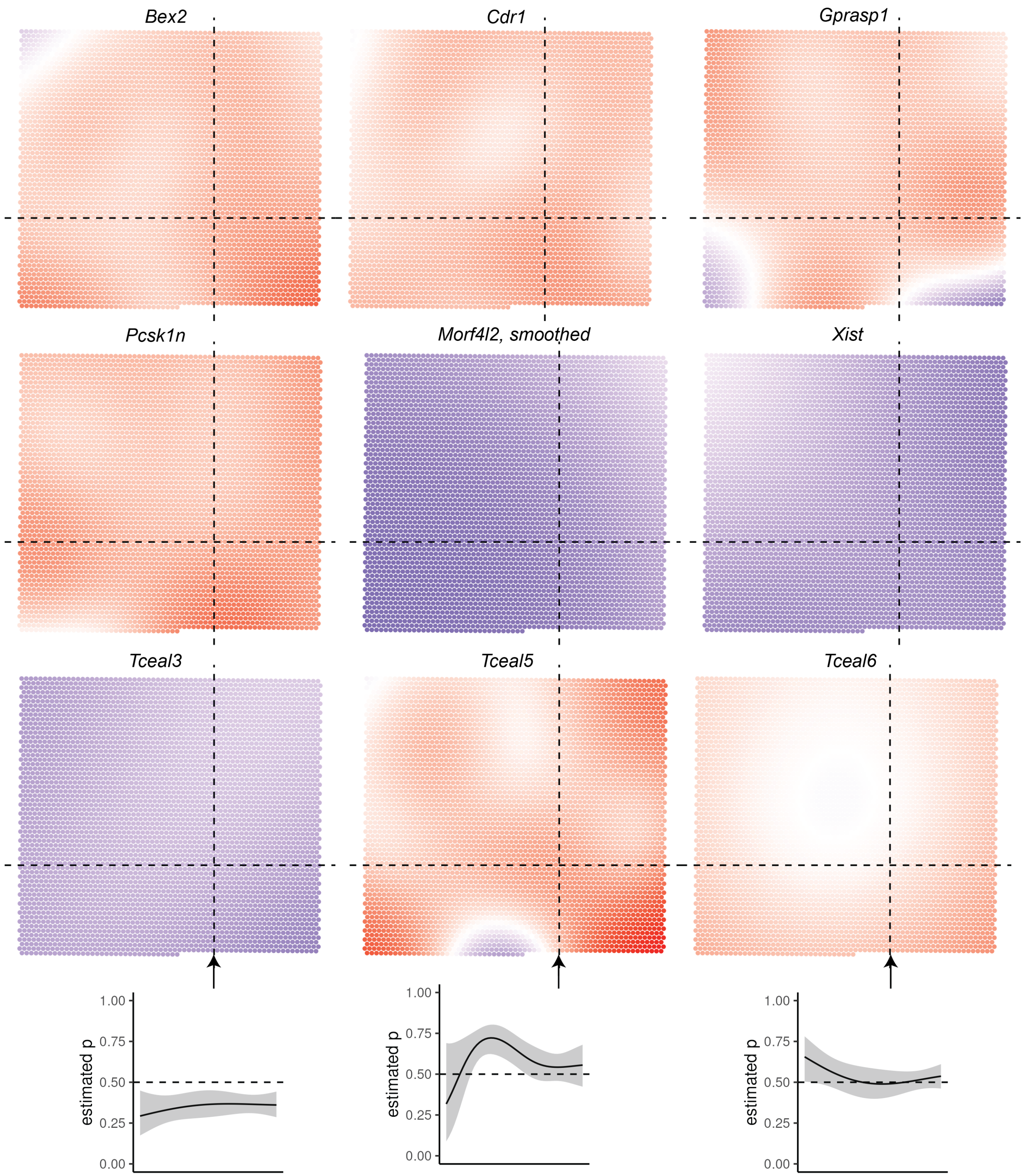

Fig. S17: Fitted 2D smooth functions for X-chromosome genes in the mouse 4 cerebellum (Visium). Confidence intervals for dotted line cross-sections are shown for *Tceal3*, *Tceal5*, and *Tceal6*.

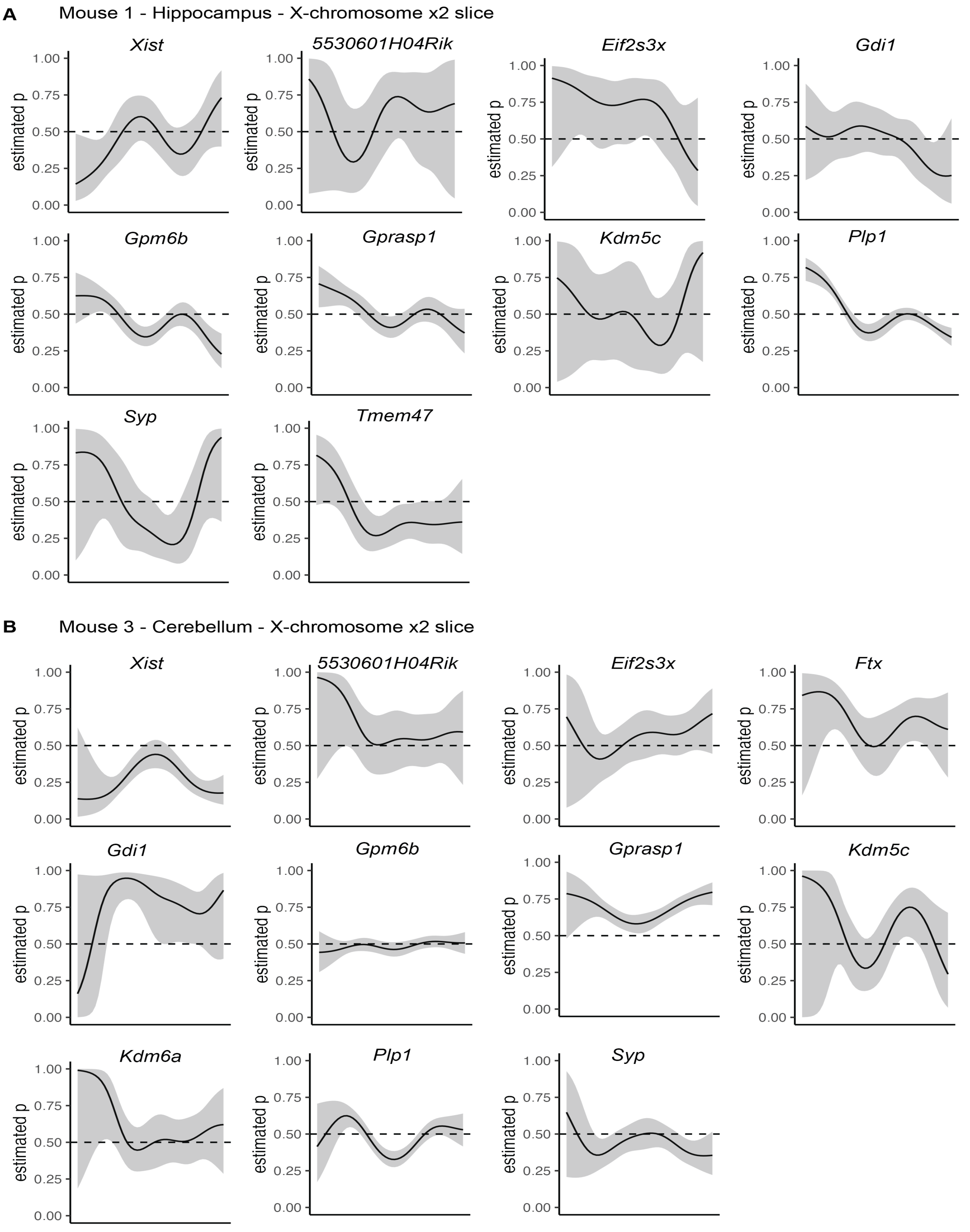

Fig. S18: Estimated maternal probability cross-sections along the x_2 direction for X-chromosome genes that were previously shown to escape XCI. **(a)** Genes fits and confidence intervals for the mouse 1 hippocampus. **(b)** Same as (a), but for the mouse 3 cerebellum.

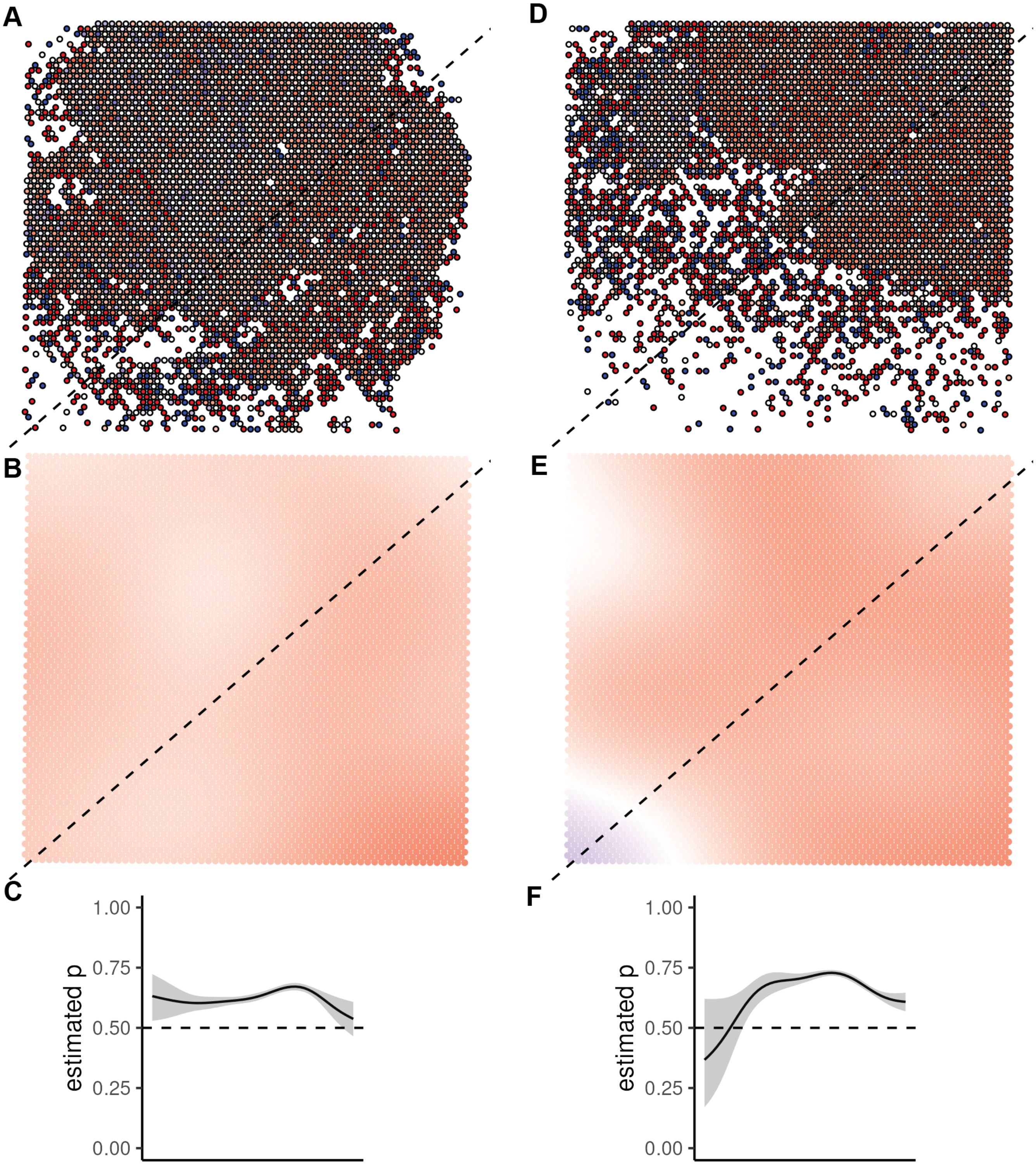

Fig. S19: Visium merged X-chromosome fits. **(a)** Raw Visium data from all (merged) X-chromosome genes, excluding *Xist*, for Mouse 4 cerebellum. **(b)** Spatial fit from non-parametric spASE. **(c)** Estimated maternal probability (estimated p) along the diagonal. **(d-f)** Sample as (a-c) but for the Mouse 5 mixture sample. Matches the spatial fits shown in Figure 4k,l, respectively.

Supplemental Notes

Additional details of the model

Here, we provide additional details on the maximum likelihood estimation procedure for spASE. spASE estimates the parameters of (2) via maximum likelihood estimation. Recall the model is as follows:

$$Y_{i,j} \sim \text{Beta-Binomial}\left\{ \sum_{k=1}^{K} \alpha_{i,j,k} \text{expit}\left(\beta_{0,k,j} + \sum_{\ell=1}^{L} \beta_{\ell,k,j} \gamma_{i,\ell} \right), N_{i,j}, \phi_j \right\}, \tag{5}$$

where $\alpha_{i,j,k}$ are pre-computed from RCTD and C-SIDE estimates. Given $\alpha_{i,j,k}$, we provide an algorithm for computing the maximum likelihood estimator of β. Our likelihood optimization algorithm is a second-order, trust-region based optimization. In brief, we iteratively solve quadratic approximations of the log-likelihood, adaptively constraining the maximum parameter change at each step. Critically, the likelihood is independent for each gene, so separate genes can be run in parallel.

Now, we consider the computation of the maximum likelihood estimator (MLE) of β for the likelihood $\mathcal{L}(\beta)$ of observing Y_i for $1 \leq i \leq I$, using the assumption that measurements on separate spots are independent. We define the predicted counts at spot i as $\bar{\lambda}_i(\beta)$, where,

$$\bar{\lambda}_i(\beta) := \sum_{k=1}^{K} \hat{\alpha}_{i,j,k} \text{expit}\left(\beta_{0,k,j} + \sum_{\ell=1}^{L} \beta_{\ell,k,j} \gamma_{i,\ell} \right). \tag{6}$$

Next, we compute the likelihood under our beta-binomial sampling model of observing Y_i counts given predicted counts $\lambda_i(\beta)$. To optimize our likelihood, we develop a second-order trust-region optimization method [52], in which sequential quadratic approximations are optimized within a trust region, whose size is determined adaptively. To do so, we first initialize β as β, which is set to 0 for intercept terms, and -5 for non-intercept terms. Additionally, we initialize the trust-region width, δ, as $\delta_0 = 0.1$. At step $n+1$ of the algorithm, with previous parameters β_n and δ_n, we make the following quadratic Taylor approximation, $\tilde{\mathcal{L}}_n$ to $\mathcal{L}$,

$$-\mathcal{L}(\beta) \approx -\tilde{\mathcal{L}}_n := -\mathcal{L}(\beta) + b(\beta)^T(\beta - \beta_n) + \frac{1}{2}(\beta - \beta_n)^T A(\beta_n)(\beta - \beta_n), \tag{7}$$

where b and A represent the gradient and Hessian of $-\mathcal{L}$, respectively, which are computed below. Next, we define β_n^* as the solution to the following optimization problem of this quadratic approximation over the trust region:

$$\begin{aligned} \min_{\beta} \quad & b(\beta_n)^T(\beta - \beta_n) + \frac{1}{2}(\beta - \beta_n)^T A(\beta_n)(\beta - \beta_n) \\ \text{s.t.} \quad & |\beta_j - \beta_{n,j}| \le \delta_n \quad \text{for} \quad 1 \le j \le \text{length}(\beta) \end{aligned} \tag{8}$$

This quadratic program is solved using the `quadprog` package in R [53]. Next, we define β_{n+1} as:

$$\beta_{n+1} := \begin{cases} \beta_n^*, & \mathcal{L}(\beta_n^*) - \mathcal{L}(\beta_n) \ge \gamma(\tilde{\mathcal{L}}_n(\beta_n^*) - \tilde{\mathcal{L}}_n(\beta_n)) \\ \beta_n, & \mathcal{L}(\beta_n^*) - \mathcal{L}(\beta_n) < \gamma(\tilde{\mathcal{L}}_n(\beta_n^*) - \tilde{\mathcal{L}}_n(\beta_n)), \end{cases} \tag{9}$$

where $\gamma = 0.1$. Additionally, the trust region is updated as:

$$\delta_{n+1} := \begin{cases} \eta_{\text{succ}}\delta_n, & \mathcal{L}(\beta_n^*) - \mathcal{L}(\beta_n) \ge \gamma(\tilde{\mathcal{L}}_n(\beta_n^*) - \tilde{\mathcal{L}}_n(\beta_n)) \\ \eta_{\text{fail}}\delta_n, & \mathcal{L}(\beta_n^*) - \mathcal{L}(\beta_n) < \gamma(\tilde{\mathcal{L}}_n(\beta_n^*) - \tilde{\mathcal{L}}_n(\beta_n)), \end{cases} \tag{10}$$

where $\eta_{\text{succ}} = 1.1$ and $\eta_{\text{fail}} = 0.5$, which, along with γ, were chosen by a combination of using standard parameter choices [52] and ensuring efficient and stable convergence to local minima. Intuitively, the quadratic approximation $\tilde{\mathcal{L}}_n$ will only be accurate within a local region, and the trust region is intended to empirically approximate that region. To test whether our local approximation is accurate, we check whether the predicted gain in log-likelihood, $\tilde{\mathcal{L}}_n(\beta_n^*) - \tilde{\mathcal{L}}_n(\beta_n)$, is close to the true gain in log-likelihood, $\mathcal{L}(\beta_n^*) - \mathcal{L}(\beta_n)$, within a factor of γ. If the local approximation is indeed accurate, the algorithm takes a step, and the trust region is allowed to grow. If not, the algorithm stays put, and the trust region shrinks. This prevents the algorithm from diverging due to poor quadratic approximations. This procedure is repeated until convergence.

Gradient and Hessian

In this section, we will derive an expression for the gradient and hessian of $-\mathcal{L}(\beta)$. First, we can calculate the gradient as,

$$\begin{aligned} b(\beta) = -\nabla L(\beta) &= -\sum_{i=1}^{I} \nabla \log Q_{Y_i}(\bar{\lambda}_i(\beta)) \\ &= -\sum_{i=1}^{I} \frac{Q'_{Y_i}(\bar{\lambda}_i(\beta))}{Q_{Y_i}(\bar{\lambda}_i(\beta))} \nabla \bar{\lambda}_i(\beta). \end{aligned} \tag{11}$$

Additionally, we have the Hessian,

$$
\begin{aligned}
A(\beta) = \text{Hess}(-L(\beta)) &= -\sum_{i=1}^{I} \nabla\left(\frac{Q'_{Y_i}(\bar{\lambda}_i(\beta))}{Q_{Y_i}(\bar{\lambda}_i(\beta))}\right)(\nabla\bar{\lambda}_i(\beta))^T - \sum_{i=1}^{I}\left(\frac{Q'_{Y_i}(\bar{\lambda}_i(\beta))}{Q_{Y_i}(\bar{\lambda}_i(\beta))}\right)\nabla^2\bar{\lambda}_i(\beta) \\
&= -\sum_{i=1}^{I}\left(\frac{Q''_{Y_i}(\bar{\lambda}_i(\beta))}{Q_{Y_i}(\bar{\lambda}_i(\beta))} - \left(\frac{Q'_{Y_i}(\bar{\lambda}_i(\beta))}{Q_{Y_i}(\bar{\lambda}_i(\beta))}\right)^2\right)(\nabla\bar{\lambda}_i(\beta))(\nabla\bar{\lambda}_i(\beta))^T \\
&\quad - \sum_{i=1}^{I}\left(\frac{Q'_{Y_i}(\bar{\lambda}_i(\beta))}{Q_{Y_i}(\bar{\lambda}_i(\beta))}\right)\nabla^2\bar{\lambda}_i(\beta).
\end{aligned} \tag{12}
$$

We use the same procedure as previously described for computing Q and its derivatives [23]. What remains is to calculate explicit expressions for $\bar{\lambda}$ and its derivatives, which we do now. Recall the definition of $\bar{\lambda}_i(\beta)$:

$$
\bar{\lambda}_i(\beta) = \sum_{k=1}^{K}\hat{\alpha}_{i,j,k}\text{expit}\left(\sum_{\ell=1}^{L_2}\gamma_{2,i,\ell}\beta_{2,\ell,k} + \sum_{\ell=1}^{L_1}\gamma_{1,i,\ell}\beta_{1,\ell}\right), \tag{13}
$$

where we now distinguish between covariates $\beta_{2,i,\ell}$ that depend on cell type k and their coefficients $\gamma_{2,i,\ell}$ and those that do not depend on cell type ($\beta_{1,\ell}$, $\gamma_{1,i,\ell}$). Next, we calculate the gradient of $\bar{\lambda}$ with respect to β_1 and β_2 separately:

$$
\begin{aligned}
\nabla_{\beta_1}\bar{\lambda}_i(\beta) &= \sum_{k=1}^{K}\hat{\alpha}_{i,j,k}\text{dexpit}\left(\sum_{\ell=1}^{L_2}\gamma_{2,i,\ell}\beta_{2,\ell,k} + \sum_{\ell=1}^{L_1}\gamma_{1,i,\ell}\beta_{1,\ell}\right)\gamma_{1,i}, \\
\nabla_{\beta_2^{(k)}}\bar{\lambda}_i(\beta) &= \hat{\alpha}_{i,k}\text{dexpit}\left(\sum_{\ell=1}^{L_2}\gamma_{2,i,\ell}\beta_{2,\ell,k} + \sum_{\ell=1}^{L_1}\gamma_{1,i,\ell}\beta_{1,\ell}\right)\gamma_{2,i},
\end{aligned} \tag{14}
$$

where we have defined $\text{dexpit}(x) = \text{expit}(x)(1 - \text{expit}(x))$. Next, we can compute the second derivatives:

$$
\begin{aligned}
\nabla_{\beta_1}\nabla_{\beta_1}\bar{\lambda}_i(\beta) &= \sum_{k=1}^{K}\hat{\alpha}_{i,j,k}\text{texpit}\left(\sum_{\ell=1}^{L_2}\gamma_{2,i,\ell}\beta_{2,\ell,k} + \sum_{\ell=1}^{L_1}\gamma_{1,i,\ell}\beta_{1,\ell}\right)\gamma_{1,i}\gamma_{1,i}^T, \\
\nabla_{\beta_2^{(k)}}\nabla_{\beta_2^{(k')}}\bar{\lambda}_i(\beta) &= \hat{\alpha}_{i,k}\text{texpit}\left(\sum_{\ell=1}^{L_2}\gamma_{2,i,\ell}\beta_{2,\ell,k} + \sum_{\ell=1}^{L_1}\gamma_{1,i,\ell}\beta_{1,\ell}\right)\gamma_{2,i}\gamma_{2,i}^T\mathbb{I}[k = k'], \\
\nabla_{\beta_2^{(k)}}\nabla_{\beta_1}\bar{\lambda}_i(\beta) &= \hat{\alpha}_{i,k}\text{texpit}\left(\sum_{\ell=1}^{L_2}\gamma_{2,i,\ell}\beta_{2,\ell,k} + \sum_{\ell=1}^{L_1}\gamma_{1,i,\ell}\beta_{1,\ell}\right)\gamma_{1,i}\gamma_{2,i}^T,
\end{aligned} \tag{15}
$$

where $\text{texpit}(x) = \text{expit}(x)(1 - \text{expit}(x))(1 - 2\text{expit}(x))$.

Finally, notice that all the above expressions, including $\bar{\lambda}_i$ and $\bar{\lambda}_i^{(k)}$ across all spots i, can be computed efficiently using matrix multiplications. Lastly, the Fisher information is computed as a scaled version of the Hessian.

Computation of Q for beta-binomial

Note, that λ is used interchangeably with p.

$$a = \lambda * (1 - \phi)/\phi$$

$$b = (1 - \lambda) * (1 - \phi)/\phi$$

$$Q_Y(\lambda) = \binom{N}{Y} \frac{B(Y + a, N - Y + b)}{B(a, b)},$$

where $B(a, b)$ is the Beta function. Set $r = (1 - \phi)/\phi$, which is constrained to be positive.

$$\log(Q_Y(\lambda)) = \log \binom{N}{Y} + \log B(Y + a, N - Y + b) - \log B(a, b)$$

$$\log(Q_Y(\lambda))' = \frac{1 - \phi}{\phi} \Big(\psi(Y + a) - \psi(N - Y + b) - \psi(a) + \psi(b) \Big)$$

$$\log(Q_Y(\lambda))'' = \left(\frac{1 - \phi}{\phi} \right)^2 \Big(\psi^{(1)}(Y + a) + \psi^{(1)}(N - Y + b) - \psi^{(1)}(a) - \psi^{(1)}(b) \Big)$$

$$\begin{aligned} \frac{\partial}{\partial r} \log(Q_Y(\lambda)) = &\lambda(\psi(Y + a) - \psi(N + a + b)) + (1 - \lambda)(\psi(N - Y + b) - \psi(N + a + b)) \\ &- [\lambda(\psi(a) - \psi(a + b)) + (1 - \lambda)(\psi(b) - \psi(a + b))] \end{aligned} \tag{16}$$

Additional notes on the spatial smoothing spline approach

The statistical properties of the smoothing spline approach have been well-established and are discussed more fully in the references we have cited in the introduction [35–39]. Here, we provide a brief summary of the intuition behind the smoothing spline method and the bias-variance tradeoff.

The spatial smoothing approach allows control over the bias-variance tradeoff via the degrees of freedom. If desired, it is possible to fit the fine-scale spatial information that is there, which amounts to estimating the spatial function with low bias, but at the cost of having a high variance around the estimate. Here, higher variance means our estimate is more susceptible to random noise and it is likely to change more if we observed a different random sample. By contrast, it is also possible to fit a function that is smoother than the true underlying pattern, with the advantage of having lower variance, at the cost of higher bias.

For illustrative purposes, we consider the 1D case (Supplemental Notes Figure S20), for which intuition generalizes to the 2D case. Suppose we have a true underlying function that describes the relationship

between spatial position and the probability of observing a maternal allele-derived transcript at that location for a particular gene. We do not observe the true function, but rather a noisy sample of observations generated from the true signal.

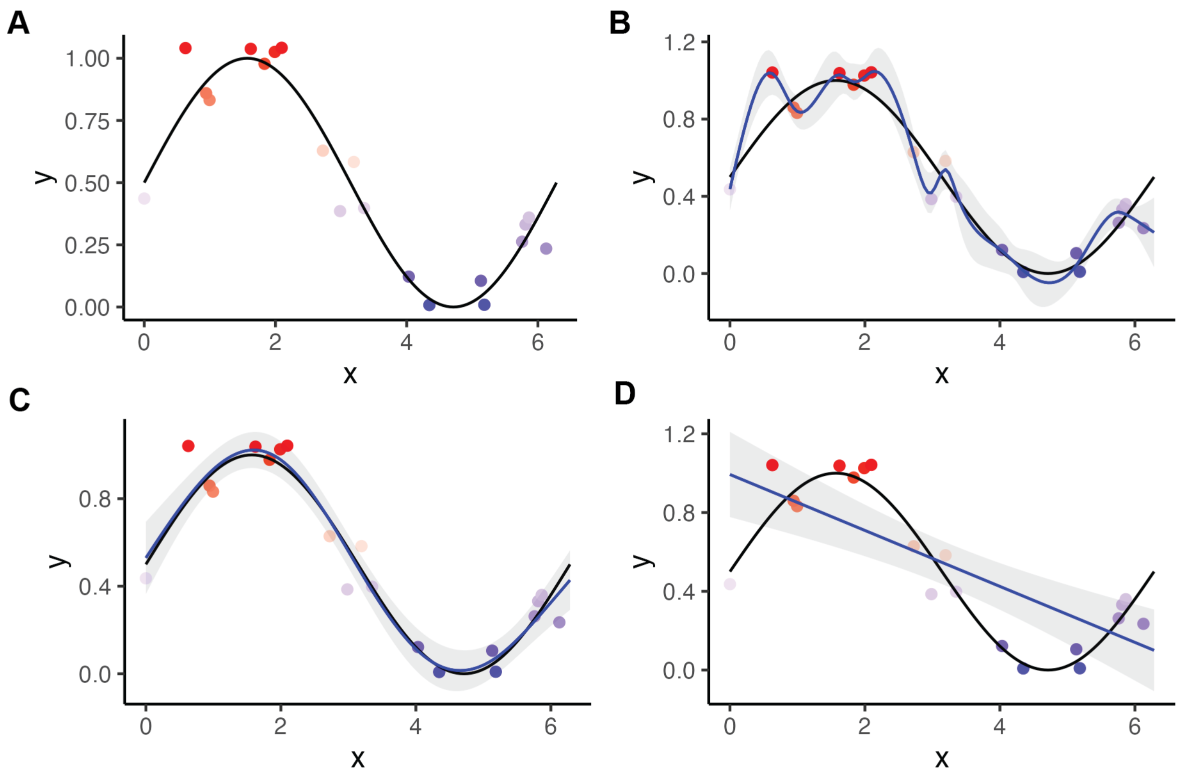

Fig. S20: Toy example in 1D of the smoothing spline approach. x represents spatial location; y represents maternal allele probability. (a) Black line indicates the ground truth, unobserved, true spatial pattern; points indicate the observations. Blue line indicates the estimated function for degrees of freedom of 15 (b), 4 (c), and 3 (d). Gray shaded region indicates 95% confidence interval around the estimate.

Suppose the black line is the true underlying function, and we have sparse, noisy observations from it (colored points, Panel A). If we wanted to fit the local behavior completely, we can fit a spline with a high degrees of freedom (15) which is close to the number of observations (20) in the data (Panel B).

Notice, however, that by fitting the local data closely, we are essentially overfitting (Panel B), i.e. fitting too much of the "random noise" induced by the data generating process. This is similar to overfitting to the random noise present in the allele-specific expression data. The estimated function is more "wiggly" than the true function.

In Panel C, we do not encourage the estimated function to "pass through" every data point as closely, but instead we force it to have some degree of smoothness by picking a lower degrees of freedom (4) (the theoretical details of which are discussed in the cited references), which gets us closer to the true underlying pattern.

In Panel D, we over-smooth; we do not capture the local curvature, but we do capture the downward trend.

In spASE, we take a two-step approach to this:

1. We first test for a significant spatial effect across all genes using low degrees of freedom (5). This lends more power to detecting significant differences. This corresponds to the tests conducted in the last two lines of Tables 1 and 2.

2. For significant genes, we then go back and estimate a spatial function with higher degrees of freedom (15). This allows us to generate higher resolution spatial maps for those genes which have sufficient numbers of spots/UMIs observed, such as those presented in Figures 3 and 4.

Our software has the flexibility to specify degrees of freedom. Raising degrees of freedom is valuable if there is a large sample size and also high resolution/density of sampled locations, such as in Slide-seq. We recommend using the same degrees of freedom when comparing spatial fits of multiple genes.
