# Supplementary material for: Detection of allele-specific expression in spatial transcriptomics with spASE
Source: Genome Biol. 2024 Jul 8;25:180. doi: 10.1186/s13059-024-03317-4 (PMC11229351; doi:10.1186/s13059-024-03317-4)
Supplement: Supplementary file 6 — Additional file 6: Review history. [file 13059_2024_3317_MOESM6_ESM.docx]

Review History

**First round of review**

**Reviewer 1**

**Were you able to assess all statistics in the manuscript, including the appropriateness of statistical tests used?**

**Yes:** Some important definitions are missing and the notations are inconsistent for describing the statistical model.

**Were you able to directly test the methods?**

No

**Comments to author:**

The research work in this manuscript investigates the spatial characteristics of allele-specific gene expressions (ASE) with a statistical approach, spASE. spASE is a beta-binomial model based on mixture of cell types and spline smoothing for spatial continuity. In simulation analysis and experiments on Visium and Slide-seq mouse brain data, examples of spatial-dependent ASEs are observed and discussed. This study focuses on the new research question of spatial ASE based on new techniques for spatial and cell-type mixture modeling. Some of the results are not clearly interpreted and the presentation still needs significant improvements. Below are some specific points.

1. The notations are inconsistent and some of the important variables/functions are undefined, which greatly hinders this reviewer's understanding of the statistical model. In particular, the spline basis functions are undefined. In equation (1) and (2), are x_i,l the knots in 2D x-y space for spline fitting? It is also confusing in Figure 1B where x_i,l is replaced with basis function B_l(X) in the non-parametric mode. What are X and B_l()? In equation (13), x is further reindexed as x_2,i,l and x_1,i,l without defining the new indexes. While it is a bit helpful to include a simple example for spline smoothing in Figure S18 in the supplementary document, a complete review of 2D spline smoothing with generalized additive model will be much more helpful for readers who are not familiar with the subject. Overall, these inconsistencies need to be reconciled and more precise notations for the background models are needed.

2. The Beta-Binomial distribution needs to be defined by the parameters. It is unclear which variant of binomial distribution in this model is and why it is an intuitive model to assume for fitting to the spatial read counts from maternal allele. Furthermore, the model given in equation (5) does not include the over-dispersion term phi_j anymore without explanation.

3. There is no description of the Visium data and the Slide-seq data used in the analysis. How many replicates are used? The pre-processing of the data is missing. In Table 1 and 2, it is unexplained how the genes are selected in the analysis (8325 in Mouse 3 Slide-seq and 10147 in Mouse 4 Visium), and these numbers are different in Tables (8319 for Mouse 3). This needs to be clarified. In addition, it is also mentioned ATAC-seq data and scRNAseq are also used in the analysis. These additional data are also unexplained.

4. More analysis is needed to justify the general applicability of the statistical modeling to real spatial transcriptomics data in Visium and Slide-seq datasets. It is unclear what the last column "Allele-resolved" in Table S1 refers to. Is this the avg. reads/spot that are allele-resolved? It seems to be a quite high ratio compared to the Avg. reads/spot in the previous column. More details of the allele-resolved read distribution per genes need to be provided. Is there sufficient support for such ASE analysis in a good number of genes?

5. Related to point (4), the number of reads in Figure 2B in simulation is at <1000, which is still way larger than the actual read counts in the real datasets. How does R^2 deteriorates in the simulation when the allele-resolved read counts is as low as those in the real data?

6. It is interesting to compare the normalized raw allele-resolved read counts with the probably computed based on the cell type deconvolution from the fitted model in Figure 3 and Figure 4. However, there is real very sparse discussion abut the cell type specificity of ASE in these genes. More annotations and systematic interpretations of the figures including Figure S2-S17 are necessary to support the claims that the cell-type specificity is playing a significant role. For example, it seems some of the spots with relatively high maternal reads are not highlighted after model fitting. Why it is happening and what is the interpretation?

**Reviewer 2**

**Were you able to assess all statistics in the manuscript, including the appropriateness of statistical tests used?**

**Yes:** The statistical methodology looks appropriate

**Were you able to directly test the methods?**

No

**Comments to author:**

In their manuscript, titled "A Novel Computational Framework for Unraveling Allelic Imbalance in Spatial Transcriptomics Datasets," Zou, Cable et al. introduce a statistical and computational framework for assessing allele-specific expression (ASE) from spatial transcriptomics data. The tool enables the identification of various potential categories of ASE, including overall allelic imbalance, cell type-specific imbalance, spatial imbalance, and cell type-specific spatial imbalance.The approach addresses technical challenges associated with single-cell RNA sequencing, including sparsity, read count overdispersion, and the variability in cell types. To quantify the contributions of each cell type to the total read counts, the authors leverage two spatial transcriptomics analysis methods previously developed by the same group that estimate cell type proportions and differential expression across spatial spots.

The computational framework is applied to transcriptome data generated from the mouse brain using two different technologies. The analysis primarily revealed genes with overall allelic imbalance and/or cell-type specific ASE, with none displaying exclusively spatial ASE. While this is probably not unexpected, the authors demonstrate that their method successfully attributes spatial ASE to variation in cell type composition across spatial spots. Importantly, the authors provide a detailed description of the limitations and scope of their method. Overall, I think this paper is a valuable contribution to the field.

Additional comments:

- The authors mention only three previously reported imprinted genes that were also identified as exhibiting ASE in their analysis. Is the direction of the imbalance (maternal/paternal) consistent with previously published results? What about other published imprinted genes? It would be useful to show whether the ones detected in the samples are found to be exhibiting ASE and if the imbalance pattern agrees with previous reports.

- The authors observe a skewed chrX inactivation in all samples with predominantly the paternal copy of the chromosome being silenced. A couple of paternally expressed genes are presented as examples of escaper genes. Are there any examples of expressed genes that display balanced expression? Do those align with previously published lists of escaper genes in mice?

- Does Fig 4C represent the estimated probability along the vertical or horizontal line of 4A?

**Authors Response**

**Point-by-point responses to the reviewers’ comments:**

Reviewer #1:

The research work in this manuscript investigates the spatial characteristics of

allele-specific gene expressions (ASE) with a statistical approach, spASE. spASE is a beta-binomial model based on mixture of cell types and spline smoothing for spatial continuity. In simulation analysis and experiments on Visium and Slide-seq mouse brain data, examples of spatial-dependent ASEs are observed and discussed. This study focuses on the new research question of spatial ASE based on new techniques for spatial and cell-type mixture modeling. Some of the results are not clearly interpreted and the presentation still needs significant improvements. Below are some specific points.

1. The notations are inconsistent and some of the important variables/functions are undefined, which greatly hinders this reviewer's understanding of the statistical model. In particular, the spline basis functions are undefined.

We use thin plate spline basis functions - we have added a section in the Methods describing this and citing the source for the implementation of thin plate splines in R:

**Non-parametric mode**

**In non-parametric mode, we implement the spline basis functions B using the thin plate spline basis [40]. To construct the thin plate spline basis functions evaluated at coordinates (x1, x2), we use the following command in R [53]:** smoothCon(s(x1,x2,k,fx=T,bs=‘tp’)) **for specified degrees of freedom k in the package** mgcv **[54].**

We have also now added additional text to clarify the notation used throughout the manuscript and have edited the notation in some places (see below).

In equation (1) and (2), are x_i,l the knots in 2D x-y space for spline fitting?

Originally, x_i,l were meant to be the lth generic user-defined covariates, which were further defined as either splines or other categorical covariates, but this was a poor choice as x was reused in later equations as the 2D spatial coordinates.

We have now removed the confusing notation of using x_i,l in Equation (1) and (2) and instead use gamma_i,l. We have added lines 105-107 to further clarify our notation introducing the model:

**“...a_i,j,k are cell type-specific weights, and the gamma_i,l represent any potential covariates of interest, such as categorical user-defined regions or a smoothing spline basis function evaluated at continuous spatial coordinates.”**

It is also confusing in Figure 1B where x_i,l is replaced with basis function B_l(X) in the non-parametric mode. What are X and B_l()?

We have now edited Figure 1 to clarify the notation as follows:

1) We have added a coordinate system in panel A showing that x_1, x_2 represent the 2D spatial coordinates
2) We have added subscript i on the bold **x** to denote the 2D spatial coordinates for spot i
3) We have added description of some of the notation at the end of the figure legend: **“Notation: Y_i,j represents the maternal allele counts at spot i for gene j. x_i=(x_1,i, x_2,i) are the 2D spatial coordinates of spot i. alpha_i,j,k are the cell type-specific weights. 1_L(.) is the indicator function for membership in set L. B_l denotes the l^th^ spline basis function.”**

In equation (13), x is further reindexed as x_2,i,l and x_1,i,l without defining the new indexes.

We have now adopted gamma instead of x here, and added additional clarification to the separation of gamma into gamma_1,i,l and gamma_2,i,l:

**“where we now distinguish between covariates beta_2,i,l that depend on cell type k and**

**their coefficients gamma_2,i,l and those that do not depend on cell type (beta_1,l, gamma_1,i,l).”**

While it is a bit helpful to include a simple example for spline smoothing in Figure S18 in the supplementary document, a complete review of 2D spline smoothing with generalized additive model will be much more helpful for readers who are not familiar with the subject.

We have added a note in the supplement that the intuition of the bias-variance trade-off that we describe in the 1D case generalizes to the 2D case, and we include references to literature that provide detailed reviews.

Overall, these inconsistencies need to be reconciled and more precise notations for the background models are needed.

See our changes above, which we believe have resolved the inconsistencies.

2. The Beta-Binomial distribution needs to be defined by the parameters. It is unclear which variant of binomial distribution in this model is

We have now added a sentence to our first results section, clarifying our parameterization of the beta-binomial distribution - see lines 101-102:

**“...where we parameterize the beta-binomial distribution in terms of mean probability, total read count, and a gene-specific overdispersion (Methods).”**

Additionally, in the Methods section, we have now added in explanation of the correspondence between our parameterization and the more familiar Beta(a,b) parameterization:

**“Note that this parameterization of the Beta distribution in terms of mean and overdispersion corresponds to a Beta(a_i,j,b_i,j) distribution where a_i,j=p_i,j*(1-phi_j)/phi_j and b_i,j=(1-p_i,j)*(1-phi_j)/phi_j”**

and why it is an intuitive model to assume for fitting to the spatial read counts from maternal allele.

We justified the use of the Beta-Binomial distribution in the 3rd paragraph of the introduction; specifically lines 48-52:

“To model [the maternal allele probability] p in bulk and single-cell RNA-seq, multiple methods have used a beta-binomial framework, which can flexibly account for overdispersion from unknown technical and biological variability. An additional advantage of this model is that it can be parameterized as a generalized linear model (GLM), allowing for maximum likelihood estimation of p while incorporating covariates of interest such as cell type.”

In the subsequent paragraph, we explain that an advantage of this framework is that this model lends itself to incorporation of covariates which may depend on spatial location, such as smoothing spline basis functions, which can be incorporated into the GLM – also termed generalized additive model.

In addition, we have cited previous work in the introduction (citations 26-28, 32) which introduce, describe, and use some variant of a beta-binomial framework successfully to model allele-specific expression in different contexts (e.g. bulk, single-cell RNA-seq).

Furthermore, the model given in equation (5) does not include the over-dispersion term phi_j anymore without explanation.

We have added phi_j into equation (5).

3. There is no description of the Visium data and the Slide-seq data used in the analysis. How many replicates are used?

We have now expanded the “Library processing” section of the Methods section to include sections on Slide-seq and Visium, which describe the data and serial sections in more detail:

**“Slide-seq**

**Slide-seqV2 was performed as described previously [21,22] on 10um-thick coronal slices of the hippocampus from three mice (Mice 1-3) and cerebellum (Mouse 3). For Mice 1 and 3, two serial sections were sequenced and reads were aggregated downstream, while for Mouse 2, only one section was sequenced. Slide-seq libraries were first processed with the slideseq-tools pipeline (**[**https://github.com/MacoskoLab/slideseq-tools**](https://github.com/MacoskoLab/slideseq-tools)**) and then re-aligned to a custom transcriptome (see below). For longer read libraries (Mouse 3 Slide-seq), we used Atropos [53] to trim adapter sequences and low quality bases.**

**Visium**

**10x Genomics Visium was performed on two mice (Mice 4-5) on 10umn-thick coronal slices of the cerebellum. Visium libraries were first processed with the 10x Genomics Space Ranger 1.1.0 pipeline and then re-aligned to a custom transcriptome (see below).“**

The pre-processing of the data is missing.

The term pre-processing is a bit non-specific: in the original manuscript, we described library processing in the Library processing section for both Slide-seq (slideseq-tools pipeline) and Visium (10x Genomics Space Ranger 1.1.0 pipeline) and also described pre-processing in terms of alignment in the Alignment section in the Methods.

We believe the reviewer also may be referring to pre-processing related to the below query about gene filtering, [etc. so](http://etc.so) we have now added additional details where relevant about certain filtering metrics that were used at each step, specifically:

1. In the “Read alignment” section of the Methods, we have added the sentence: **“We merged the reads from samples that had two serial sections: Mouse 1 hippocampus, Mouse 3 hippocampus, and Mouse 3 cerebellum.”**
2. In the “Cell type assignment” section of the Methods, we have added in additional information about the settings used for the RCTD step: **“We ran RCTD in doublet mode for the Slide-seq samples (default settings: MIN.CHANGE = 0.001, CONFIDENCE_THRESHOLD = 10, DOUBLET_THRESHOLD = 25) and full mode for the Visium samples (default settings: MIN.CHANGE = 0.001).”**
3. In the “Cell type-specific differential expression” section of the Methods, we have added in additional information about the settings used for the C-SIDE step: **“We ran C-SIDE as previously described in non-parametric mode, filtering to gene and cell type combinations with at least 128 spots with non-zero counts.”**

In Table 1 and 2, it is unexplained how the genes are selected in the analysis (8325 in Mouse 3 Slide-seq and 10147 in Mouse 4 Visium),

We have now added a sentence in the legend of Table 1 specifying:

**“The total number of genes for each sample is based on using a filtering threshold of at least 128 spots with non-zero counts per gene.”**

and these numbers are different in Tables (8319 for Mouse 3). This needs to be clarified.

Note that Table 1 describes the cerebellum results and Table 2 describes the hippocampus, which are two distinct samples for Mouse 3. This explains why the numbers are slightly different after the

filtering threshold. However, the fact that they are so close reflects the observation we made in lines 166-168, which is that the two different regions of Mouse 3 were highly concordant. Also note: we have slightly changed these numbers in a re-analysis (8,304 and 8,309).

In addition, it is also mentioned ATAC-seq data and scRNAseq are also used in the analysis. These additional data are also unexplained.

We have added the following sections to the end of the Methods section (see manuscript for citations):

**“Mouse hippocampus scATAC-seq, cis-regulatory elements, and TFBS motifs**

**We analyzed previously published mouse hippocampus sci-ATAC-seq count matrices (GSE118987). The mice used in the study were the wild-type C57/B6 strain, and the data were aligned to the mm10 genome. We extracted the called peak annotations and counts using the command scitools split. To quantify accessibility of peaks, we computed the average count within cell types. We searched for known TFBS motifs within peaks with the command matchMotifs from the R package motifmatchr using all motifs for Mus musculus in the JASPAR 2020 database. We overlaid annotations of ENCODE cis-regulatory elements (cCREs) for mm10 downloaded from the UCSC Table Browser at the *Ptgds* locus and visualized the annotations using IGV.**

**Mouse strain SNP data**

**We obtained gene-specific SNP annotations for 129S1/SvImJ and CAST/EiJ with respect to the mm10 reference using REL1505 of the Mouse Genomes Project (**[**https://www.sanger.ac.uk/sanger/Mouse_SnpViewer/rel-1505).**](https://www.sanger.ac.uk/sanger/Mouse_SnpViewer/rel-1505).)

**Mouse scRNA-seq data**

**We downloaded mouse scRNA-seq data from the Allen Brain Map, filtered to cell types annotated that were present in our mouse hippocampus data set, and visualized the expression of *Mtf1*.”**

4. More analysis is needed to justify the general applicability of the statistical modeling to real spatial transcriptomics data in Visium and Slide-seq datasets. It is unclear what the last column "Allele-resolved" in Table S1 refers to. Is this the avg. reads/spot that are allele-resolved?

We have now added a sentence in the Table S1 legend clarifying:

**“Allele-resolved is the average number of reads per spot that were able to be uniquely assigned to one allele.”**

It seems to be a quite high ratio compared to the Avg. reads/spot in the previous column.

Note that we are using an F1 hybrid mouse strain which is not typically used in most experiments. As we describe in the Methods section “CAST/EiJ x 129S1/SvImJ F1 mice”, this F1 hybrid has a high SNP density between the two parental strains, which allows for a high rate of allele-resolved transcripts.

More details of the allele-resolved read distribution per genes need to be provided. Is there sufficient support for such ASE analysis in a good number of genes?

To provide further details on the allele-resolved read distribution, we have now added a new Supplemental Figure S2 which shows the histogram of total read counts per gene as well as total allele-resolved read counts per gene (replicated here for convenience):

The red dashed line shows our cutoff of a minimum of 2^7 (128) counts per gene. (Also, see above response to point (3) where we now mention that we filtered to genes with at least 128 allele-resolved counts across all spots). This filter left us a range of ~2,500-10,000 genes to analyze, depending on the coverage of the sample (total n genes in Tables 1 and 2) - we believe this is a good amount of genes.

5. Related to point (4), the number of reads in Figure 2B in simulation is at <1000, which is still way larger than the actual read counts in the real datasets. How does R^2 deteriorates in the simulation when the allele-resolved read counts is as low as those in the real data?

We have now modified Figure 2B to include ranges below 1,000 counts. We now bin into <100, 100-500, 500-1,000, 1,000-5,000, 5,000-10,000, >10,000. We have also changed the visualization from R^2 to just Pearson correlation r, to allow plotting of instances where there was negative correlation. We have also included the following text in the Results:

**“Even at 100-500 counts, correlation was still weakly positive, with the IQR of the boxplots generally lying above r=0.5. This motivated our selection of the threshold of 2^7 (128 counts) in analyses using real data, as well as using confidence intervals over the 2D surface to assess the variance in the estimated 2D spatial function and account for cases where the density of counts for a particular gene was not high enough to make an accurate estimate.”**

In addition, we also changed panel D to include only results from the 100-500 range, and to include results from all cell types, not just Granule cells:

6. It is interesting to compare the normalized raw allele-resolved read counts with the probably computed based on the cell type deconvolution from the fitted model in Figure 3 and Figure 4. However, there is real very sparse discussion abut the cell type specificity of ASE in these genes.

We now have added more text and figures into the Results to elaborate on the cell type specificity of ASE:

1. We added panel H in Figure 3 to further emphasize that these results were driven by cell type, plotting the point estimate and 95% confidence interval around the point estimate for each cell type for *Ptgds* and *Sst*:

2. We also added panel I in Figure 3 which show histograms of the raw maternal proportion per spot for Sst, showing that the high maternal expression and spatial pattern is most strongly due to the Interneuron subtype:

3. We have expanded the text of the Results significantly:

**“For example, consider the gene Ptgds, which exhibited significant overall spatial ASE pattern of a general paternal bias with a small region of biallelic expression in the mouse hippocampus (Figure 3a-c). These regions corresponded to the localization of two major cell types which highly expressed Ptgds: oligodendrocytes and endothelial cells. When using spASE to estimate the effect size of ASE within cell types, we found that oligodendrocytes had a strong paternal bias in expression, whereas endothelial cells had strong biallelic expression (Figure 3h). When using the full spASE model controlling for cell type, Ptgds was not significant for having a within cell type spatial ASE effect. Thus, we conclude that Ptgds exhibits a cell type-driven spatial ASE effect due to the contrast between the cell type-specific biallelic expression in endothelial cells and high paternal expression in oligodendrocytes. We wondered what potential mechanism could be driving the paternal bias in oligodendrocyte expression (Figure 3h). Integrating single-cell ATAC-seq data and single-cell RNA-seq atlas data, we found a likely mechanism by which an oligodendrocyte-specific enhancer for Ptgds may have a strain-specific SNP which alters an oligodendrocyte-specific TF binding site (Supplemental Figure S12).**

**We also discovered a cell type-driven spatial effect in Sst in the hippocampus. In particular, we noticed a strong maternal bias in the bottom-middle region of the puck, corresponding to a cluster of interneurons outside of the main hippocampal formation, in the thalamus (Figure 3e-g). Indeed, when we fit the intercept-only spASE model and examined the estimated maternal probabilities within cell type, we found that this interneuron subtype had a higher maternal estimated maternal probability that was further from 50/50 than other interneurons (Figure 3h). This bias was also visible in the distributions of raw maternal proportion of total reads per cell type (Figure 3i). Thus, the localization of this interneuron subtype to a small region of the puck, combined with its high expression of Sst, was the main driver of the spatial pattern in Sst.”**

More annotations and systematic interpretations of the figures including Figure S2-S17 are necessary to support the claims that the cell-type specificity is playing a significant role.

We believe the above additional text and figures now support the claims that cell-type specificity is playing a significant role, and have referenced the supplemental figures where appropriate (e.g. Figures S13 onward are related to the X-chromosome analysis and not the cell type-specificity analysis, and so are not mentioned).

For example, it seems some of the spots with relatively high maternal reads are not highlighted after model fitting. Why it is happening and what is the interpretation?

To clarify how to interpret the spots with “relatively high maternal reads”, we have modified the raw data plots in Figure 3, panels b and f. Specifically, we now use the size of the point to show how many total counts are present at that spot. This visualization makes it more obvious that many of the spots with a high maternal fraction in fact have a low total allele-resolved count.

This new visualization should emphasize the fact that low total counts result in a noisy maternal fraction at that spot in the raw data.

**Reviewer #2:**

In their manuscript, titled "A Novel Computational Framework for Unraveling Allelic Imbalance in Spatial Transcriptomics Datasets," Zou, Cable et al. introduce a statistical and computational framework for assessing allele-specific expression (ASE) from spatial transcriptomics data. The tool enables the identification of various potential categories of ASE, including overall allelic imbalance, cell type-specific imbalance, spatial imbalance, and cell type-specific spatial imbalance.The approach addresses technical challenges associated with single-cell RNA sequencing, including sparsity, read count overdispersion, and the variability in cell types. To quantify the contributions of each cell type to the total read counts, the authors leverage two spatial transcriptomics analysis methods previously developed by the same group that estimate cell type proportions and differential expression across spatial spots.

The computational framework is applied to transcriptome data generated from the mouse brain using two different technologies. The analysis primarily revealed genes with overall allelic imbalance and/or cell-type specific ASE, with none displaying exclusively spatial ASE. While this is probably not unexpected, the authors demonstrate that their method successfully attributes spatial ASE to variation in cell type composition across spatial spots. Importantly, the authors provide a detailed description of the limitations and scope of their method. Overall, I think this paper is a valuable contribution to the field.

Additional comments:

- The authors mention only three previously reported imprinted genes that were also identified as exhibiting ASE in their analysis. Is the direction of the imbalance (maternal/paternal) consistent with previously published results?

The three we mentioned in the original manuscript were *Meg3* (maternal), *Impact* (paternal), and *Peg3* (paternal), all of which agreed with previous reports - this is now stated where referenced in lines 156-157:

**“...including *Meg3*, *Impact*, and *Peg3*, for which our estimated direction of ASE bias was consistent with previous reports as maternal (*Meg3*) and paternal (*Impact*, *Peg3*) imprinted genes [43].”**

What about other published imprinted genes? It would be useful to show whether the ones detected in the samples are found to be exhibiting ASE and if the imbalance pattern agrees with previous reports.

To address this, we have now added:

1. In Supplemental Tables S2 (overall maternal or paternal bias per gene) and S3 (within cell type maternal or paternal bias), we have added three additional columns: 1) whether or not the gene is present in a list of published imprinted genes from Santini et al., 2021, which has imprinted status but not the direction of ASE bias; 2) whether or not the gene is listed as imprinted on <https://geneimprint.org> ; and 3) the direction of ASE bias from <https://geneimprint.org> .

2. Additional text in the results section, elaborating on these results:

**“We compared these estimates to a previously published list of 383 imprinted genes in mice (not specific to the brain) [44] and found that 91 of them were also detected as imprinted in our data (Supplemental Tables S2, S3). We also used a resource which contains previous knowledge on the direction of ASE effect for 138 genes (not specific to the brain) [43] and found that, of the 35 genes estimated to have ASE in our data set, 26 of them (18 paternal and 8 maternal) agreed with the previous direction of effect (Supplemental Tables S2, S3).”**

3. A summary paragraph of these findings in the Discussion:

**“Overall, we found modest agreement between the imprinted genes reported we report here, but many of the genes we found were not in any previously published list of imprinted genes [43, 44]. In particular, only 91 of 383 previously published imprinted genes were detected in our study. However, of the genes that had previously published direction of ASE and that were detected as significant in our analysis, 26 of 35 agreed in the direction of ASE. There are many possible explanations for these differences, including 1) our study was focused on the mouse cerebellum and hippocampus, whereas many studies have not focused on these tissues, and many genes have previously been shown to have imprinting dependent on the tissue of interest [43]; and 2) the genetic background of our mouse model could potentially alter some imprinted directions, particularly with the abundance of SNPs that could affect gene regulatory regions.”**

- The authors observe a skewed chrX inactivation in all samples with predominantly the paternal copy of the chromosome being silenced. A couple of paternally expressed genes are presented as examples of escaper genes. Are there any examples of expressed genes that display balanced expression?

Yes, we found that *Gpm6b* and *Syp* that had evidence of balanced expression - see our added text to the Results below.

Do those align with previously published lists of escaper genes in mice?

We have now added text to the Results and Discussion about this, as well as a **new Supplementary Figure S19** highlighting the escaper genes:

In the Results:

**We then looked for any genes potentially escaping X-chromosome inactivation (XCI). We compared the estimated maternal probability functions for a previously published list of 14 XCI escape genes in the mouse brain [45], and found that 11 (including Xist) were highly expressed enough in our data to enable estimation of spatial ASE. Of those 11, Gpm6b and Syp showed evidence of XCI escape in the cerebellum, but not the hippocampus. In particular, Gpm6b had near-biallelic expression across the slice, had a spatial function that was similar, although more attenuated in magnitude, to other X-chromosome genes that did not escape XCI. Syp also exhibited near-biallelic expression, and exhibited a similar spatial pattern to Xist (Supplemental Figure S19). The other genes either had too few counts to confidently estimate a spatial pattern with a small confidence interval, or they appeared to not escape XCI. We also found evidence of XCI escape genes not previously reported; for example, Morf4l2 showed a strong paternal bias across all five mice and both brain regions. In mice 1 and 4, Tceal3 appeared to have a strong paternal bias, and similarly,Tceal6 had a pattern that trended closer to biallelic expression than other X-chromosome genes, potentially indicating escape from XCI.**

**Figure S19**: Estimated maternal probability cross-sections along the x2 direction for X-chromosome genes that were previously shown to escape XCI. (a) Genes fits and confidence intervals for the mouse 1 hippocampus. (b) Same as (a), but for the mouse 3 cerebellum.

In the Discussion:

**Interestingly, we found that Gpm6b and Syp, two genes previously published to escape XCI in the brain [45], exhibited XCI escape in the cerebellum but not the hippocampus, and many of the previously reported genes did not escape XCI in our data. This could point to tissue- or sample-specific differences in XCI. One limitation was that many of the previously reported genes were not measured at high enough read depth in our data, and thus had very wide confidence intervals across the spatial fit (Supplemental Figure S19).**

- Does Fig 4C represent the estimated probability along the vertical or horizontal line of 4A? It is along the vertical line; we have now added additional clarification in the figure legend:

**“(c) Estimate and confidence interval along the vertical dashed line in a, starting at the arrow in panel a and moving up.”**

**Second round of review**

**Reviewer 1**

The authors have addressed most of my comments in this revision. Below are two remaining points to consider,

1. While the clarity of the model description is drastically improved in this revision, what remains obscure is the choice of the k knots in the non-parametric mode. The section "Non-parametric mode" on page 19 is not helpful without any providing any detail about how k is decided and what are the resulting k knots. Is the k the number of data points as for smoothing spline or k is tuned for different data/platform such as those for Visium data and Slide-seq and consideration of spatial patterns. In the experiment testing varying k in Figure S12-17, it is unstated which k are tested and how they selected. It appears different k were used in different datasets. There need be a detailed discussion in the method about the choice of k and how the k knots are selected.

2. In the description in Table 1 & 2, genes expressed at at least 128 spots were analyzed but the cutoff in Figure S2 was for 128 read counts. The cutoff line should be corrected in Figure S2.

**Reviewer 2**

The authors have added the analyses I requested: no further suggestions.

**Authors Response**

**Point-by-point responses to the reviewers’ comments:**

Reviewer #1:

The authors have addressed most of my comments in this revision. Below are two remaining points to consider,

1. While the clarity of the model description is drastically improved in this revision, what remains obscure is the choice of the k knots in the non-parametric mode. The section "Non-parametric mode" on page 19 is not helpful without any providing any detail about how k is decided and what are the resulting k knots. Is the k the number of data points as for smoothing spline or k is tuned for different data/platform such as those for Visium data and Slide-seq and consideration of spatial patterns. In the experiment testing varying k in Figure S12-17, it is unstated which k are tested and how they selected. It appears different k were used in different datasets. There need be a detailed discussion in the method about the choice of k and how the k knots are selected.

*In non-parametric mode, we stated that k is the degrees of freedom for the thin plate spline basis function: “...for specified degrees of freedom k in the packages mgcv.”*

*We now include additional sentences under “Non-parametric mode” detailing our choices of k:*

*For both Slide-seq and Visium data, we experimented with multiple choices of k, and selected k=5 for testing significant spatial patterns (Tables 1 and 2). We used k=15 to provide higher resolution visualizations for genes with a significant spatial pattern (Figures 3, 4, and Supplemental Figures S12-17; see supplemental note for additional information on the intuition of adjusting k).*

2. In the description in Table 1 & 2, genes expressed at at least 128 spots were analyzed but the cutoff in Figure S2 was for 128 read counts. The cutoff line should be corrected in Figure S2.

*We have now removed the cutoff line as Figure S2 is just meant to show the distributions of read counts, and does not include information about the number of spots.*
